# Obstetric and perinatal outcomes in women with previous breast cancer: a nationwide study of singleton births 1973–2017

**DOI:** 10.1093/hropen/hoae027

**Published:** 2024-05-04

**Authors:** Leo Gkekos, Anna L V Johansson, Kenny A Rodriguez-Wallberg, Irma Fredriksson, Frida E Lundberg

**Affiliations:** Department of Medical Epidemiology and Biostatistics, Karolinska Institutet, Stockholm, Sweden; Department of Medical Epidemiology and Biostatistics, Karolinska Institutet, Stockholm, Sweden; Cancer Registry of Norway, Norwegian Institute of Public Health, Oslo, Norway; Department of Oncology-Pathology, Laboratory of Translational Fertility Preservation, Karolinska Institutet, Stockholm, Sweden; Division of Gynecology and Reproduction, Department of Reproductive Medicine, Karolinska University Hospital, Stockholm, Sweden; Department of Molecular Medicine and Surgery, Karolinska Institutet, Stockholm, Sweden; Department of Breast, Endocrine Tumors and Sarcoma, Karolinska University Hospital, Stockholm, Sweden; Department of Medical Epidemiology and Biostatistics, Karolinska Institutet, Stockholm, Sweden; Department of Oncology-Pathology, Laboratory of Translational Fertility Preservation, Karolinska Institutet, Stockholm, Sweden

**Keywords:** breast cancer, delivery, obstetric, perinatal, pregnancy, breast cancer survivor

## Abstract

**STUDY QUESTION:**

What are the obstetric and perinatal outcomes in births to breast cancer survivors compared to women without previous breast cancer?

**SUMMARY ANSWER:**

Women who conceived during the first 2 years following a breast cancer diagnosis had a higher risk for preterm birth, induced delivery, and cesarean section, while no increased risks were observed in births conceived later than 2 years after a breast cancer diagnosis.

**WHAT IS KNOWN ALREADY:**

A recent meta-analysis found higher risks of cesarean section, preterm birth, low birthweight, and small for gestational age in pregnancies among breast cancer survivors. Less is known about rarer outcomes such as pre-eclampsia or congenital malformations.

**STUDY DESIGN, SIZE, DURATION:**

We conducted a population-based matched cohort study including all breast cancer survivors who gave birth to singletons 1973–2017 in Sweden, identified through linkage between the Swedish Cancer Register, the Medical Birth Register, and the National Quality Register for Breast Cancer.

**PARTICIPANTS/MATERIALS, SETTINGS, METHODS:**

Each birth following breast cancer (n = 926) was matched by maternal age at delivery, parity, and calendar year at delivery to 100 births in a comparator cohort of women (n = 92 490). Conditional logistic and multinomial regression models estimated relative risks (RR) with 95% CI. Subgroup analyses by time since diagnosis and type of treatment were performed.

**MAIN RESULTS AND THE ROLE OF CHANCE:**

Previous breast cancer was associated with higher risks of induced delivery (RR; 1.3, 1.0–1.6), very preterm birth (RR; 1.8, 1.1–3.0), and planned preterm birth (RR; 1.6, 1.0–2.4). Women who conceived within 1 year after breast cancer diagnosis had higher risks of cesarean section (RR; 1.7, 1.0–2.7), very preterm birth (RR; 5.3, 1.9–14.8), and low birthweight (RR; 2.7, 1.4–5.2), while the risks of induced delivery (RR; 1.8, 1.1–2.9), moderately preterm birth (RR; 2.1, 1.2–3.7), and planned preterm birth (RR; 2.5, 1.1–5.7) were higher in women who conceived during the second year after diagnosis. Women who conceived later than 2 years after breast cancer diagnosis had similar obstetric risks to their comparators.

**LIMITATIONS, REASONS FOR CAUTION:**

As information on the end date of treatment was unavailable, the time between the date of diagnosis and conception was used as a proxy, which does not fully capture the effect of time since end of treatment. In addition, treatments and clinical recommendations have changed over the long study period, which may impact childbearing patterns in breast cancer survivors.

**WIDER IMPLICATIONS OF THE FINDINGS:**

Risks of adverse obstetric outcomes in breast cancer survivors were confined to births conceived within 2 years of diagnosis. As family building holds significance for numerous young breast cancer patients, these findings are particularly important to inform both breast cancer survivors and clinicians about future reproductive outcomes.

**STUDY FUNDING/COMPETING INTEREST(S):**

This work was supported by the Swedish Cancer Society (grant number 22-2044 Pj A.L.V.J.), Karolinska Institutet Foundations (grant number: 2022-01696 F.E.L., 2022-01559 A.L.V.J.), and the Swedish Research Council (grant number: 2021-01657 A.L.V.J.). K.A.R.-W. is supported by grants from the Swedish Cancer Society (20 0170 F) and the Radiumhemmets Research Foundations for clinical researchers 2020–2026. The authors declare that they have no conflicts of interest.

**TRIAL REGISTRATION NUMBER:**

N/A.

WHAT DOES THIS MEAN FOR PATIENTS? In Europe, around one-third of breast cancers occur before age 50 years, and it is the most common malignancy in women under 35 years of age. As the number of younger breast cancer survivors who wish to conceive is increasing, clinicians are asked several questions about future fertility, maternal health, and potential risks to their offspring. Several studies have found higher risks of cesarean section, preterm birth, low birthweight, and small for gestational age in pregnancies among breast cancer survivors, but only a few studies have been able to investigate rarer events, such as pre-eclampsia or malformations. We investigated all 926 singleton births to breast cancer survivors in Sweden between 1973 and 2017. We found that women with a previous breast cancer who gave birth were more likely to have an induced delivery, very preterm birth, and planned preterm birth compared to women without previous cancer. The higher risks of adverse obstetric outcomes were confined to births conceived within the first 2 years following a breast cancer diagnosis. Since family building is important to many young breast cancer patients, these findings are particularly important to inform breast cancer survivors and clinicians about future reproductive outcomes.

## Introduction

Around one-third of breast cancers occur before the age of 50 years (Forouzanfar *et al.*, 2011; Arnold *et al.*, 2022), and it is the most common malignancy in women under 35 years of age (Radecka and Litwiniuk, 2016). Improved survival rates and changes in reproductive patterns, particularly postponing childbearing, have highlighted several issues for women who wish to conceive after cancer (Cardoso *et al.*, 2012; Hill *et al.*, 2018). Concerns regarding future infertility and the ability to reproduce have been identified as important issues for young breast cancer survivors (Stan *et al.*, 2013). Clinicians are increasingly confronted with questions about the safety of pregnancy after intense cancer treatment, both with respect to adverse maternal outcomes and the impact on the infant.

A recent meta-analysis found higher risks of cesarean section, preterm birth, low birthweight, and small for gestational age in pregnancies among breast cancer survivors (Lambertini *et al.*, 2021). Only a few studies have been able to investigate less common outcomes, such as the risk of pre-eclampsia (Jacob *et al.*, 2017; Lee *et al.*, 2019), prelabor rupture of membranes (Lee *et al.*, 2019), or congenital malformations (Dalberg *et al.*, 2006; Langagergaard *et al.*, 2006; Stensheim *et al.*, 2013), with conflicting results. In addition, time since breast cancer diagnosis has not been explored in relation to these rarer outcomes.

This population-based study aims to provide a comprehensive overview of adverse obstetric and perinatal outcomes in births of breast cancer survivors compared to cancer-free population comparators and to investigate whether the risks of outcomes differ by time between breast cancer diagnosis and conception, calendar period of diagnosis, or by type of breast cancer treatment received.

## Materials and methods

### Study design and study population

This is a population-based matched cohort study, where individual-level linkages between registers were performed via the personal identification number assigned to all Swedish residents. The study base consisted of births to women who were born 1943–1999 and recorded in the Swedish Multigeneration Register at Statistics Sweden. Births of these women between 1973 and 2017 at ages 18–49 years were identified in the Swedish Medical Birth Register (MBR) (N = 4 638 397) (Supplementary Fig. S1). Cancer diagnoses of the women between 1958 and 2017 were identified in the Swedish Cancer Register (SCR). Births of women with reused personal identity numbers (N = 81) were excluded. Multiple births (N = 115 596) were also excluded because of their inherent higher obstetrical and perinatal risks (Santana *et al.*, 2018). Births of women with cancer other than breast cancer diagnosed before (N = 15 879) or during pregnancy (N = 1094) and of women with breast cancer during pregnancy (N = 222) were also excluded. Births of women with previous breast cancer (n = 926) were matched to comparators (births of women without a history of cancer) in a ratio of 1:100, with the intent of capturing some of the rarer outcomes in the comparator group, such as congenital malformations, which have a low prevalence of 2–3% in the general population (Corsello and Giuffrè, 2012). The matching variables included maternal age at delivery (±3 months in women aged 18–42 years, 12 months in ages 43–44 years, and 36 months in ages 45–49 years), parity (first, second, and third or higher birth order of the child) and calendar time at delivery (in 5-year periods). The distribution of age and year of delivery among women with previous breast cancer and matched comparators is shown in Supplementary Fig. S2. Information on the women’s country of birth and education level at the year of delivery was obtained from Statistics Sweden. Data on maternal height and pre-pregnancy weight, as well as self-reported information on smoking during pregnancy, infertility, and use of fertility treatments to conceive (either through ovulation induction, IVF, or ICSI), were obtained from the MBR.

### Obstetric and perinatal outcomes

The MBR was established in 1973 and includes detailed clinical information on all deliveries in Sweden from gestational week 28 (1973–June 2008) and thereafter from gestational week 22 (from July 2008 and onwards). Pregnancies ending in miscarriages or abortions are not registered in the MBR. The completeness of MBR is very high with only 1–3% of births being missed per year over the past 20 years (Cnattingius *et al.*, 2023). Included obstetric and perinatal outcomes from MBR were gestational diabetes, gestational hypertension, pre-eclampsia, placental abruption, bleeding during pregnancy, prelabor rupture of membranes, cesarean section, birth injury, stillbirth, gestational age (categorized as very preterm: <32^+0 ^weeks, moderately preterm: 32^+0^–36^+6 ^weeks, and term: ≥37^+0 ^weeks), birthweight, Apgar score at 5 min, major and minor malformations, and neonatal mortality (0–27 days after delivery) (Supplementary Table S1). Information on induced delivery and delivery mode (unassisted or assisted vaginal, planned, or emergency cesarean) was available from 1990. Planned preterm birth was defined as either induced delivery or planned cesarean section before 37^+0 ^weeks of gestation. Birthweight for gestational age in percentiles was calculated according to Swedish sex-specific standard growth curves (Lindström *et al.*, 2021).

### Tumor characteristics

The SCR, established in 1958, includes information such as date of diagnosis, tumor localization, and morphological diagnosis of tumors. Cancer diagnoses in the SCR are coded using the International Classification of Diseases Oncology version 3 (ICD-O3), and back-translated to ICD-7 to enable comparisons over time. Comprehensive quality assurances and morphological verification of each cancer diagnosis are practices that ensure the high quality of the register, which has a reported completeness of up to 99% (Barlow *et al.*, 2009). We included diagnoses of invasive breast cancer (ICD-7: 170) from the SCR. In addition, the historical Regional Breast Cancer Quality Registers 1992–2007, and the National Quality Register for Breast Cancer 2008–2017 were used to acquire detailed clinical breast cancer information (Fredriksson, 2023). The breast cancer registers include information on breast cancer diagnoses (ICD-10: C50) in Sweden and has national coverage since 1992. We included information on estrogen receptor (ER) receptor, human epidermal growth factor receptor 2 (HER2), and progesterone receptor (PR) status, as well as clinical and pathological tumor size, nodal status and metastatic disease, and information on surgical and oncological treatment (type of surgery, chemotherapy, radiotherapy, endocrine therapy, and anti-HER2-treatment). The breast cancer registers have very high completeness with a coverage across regions and years of >99% (Löfgren *et al.*, 2019).

### Statistical analyses

The study design was a population-based matched cohort study, where the exposure was maternal breast cancer, and the cohort was assessed cross-sectionally at birth for the birth outcomes. Obstetric and perinatal outcomes of exposed pregnancies were compared to those of pregnancies in women without previous breast cancer using conditional logistic and multinomial regression, estimating odds ratios (ORs) and relative risk ratios (RRRs) with 95% CI. Based on the rare disease assumption, both were denoted as relative risks (RRs). The presented models were conditioned on the matched sets (maternal age, parity, and calendar time at delivery), and additionally adjusted for maternal country of birth. All analyses were performed on complete cases, where births missing outcome information were excluded from the respective model (Supplementary Table S2). A sensitivity analysis was performed, further adjusting for highest attained education, pre-pregnancy BMI and smoking during pregnancy, after using multiple imputation by chained equations to account for missing values of these covariates (White *et al.*, 2011; StataCorp. 2023. Stata 18 Multiple-Imputation Reference Manual. College Station, TX, USA: Stata Press).

As secondary analyses, first, we assessed the outcomes stratified by two time periods (1960–1997, 1998–2016) of breast cancer diagnosis. Second, we assessed the outcomes stratified by time from breast cancer diagnosis to conception (<1, 1–<2, 2–<3, 3–<5, and ≥5 years). Third, we assessed the outcomes within the subsets of women who either underwent chemotherapy or did not, stratified by time since breast cancer diagnosis (<2 and ≥2 years). Secondary analyses were also conditioned on the matched sets, i.e. each exposed birth was matched to its own subset of unexposed. Likelihood-ratio tests were performed to assess the interactions of the secondary analyses. We used a significance level of 5%. All analyses were carried out in Stata (StataCorp. 2023. Stata Statistical Software: Release 18. College Station, TX, USA: StataCorp LLC).

### Ethical approval

The study was performed without informed consent under ethical approval by the Swedish Ethical Review Board (reference number 2010-1950-31/4), with amendments (2011-599-32, 2018/1293-32, 2022-02992-02) approved by the Swedish Ethical Review Authority.

## Results

We identified 926 births to breast cancer survivors who delivered a child between 1973 and 2017 in Sweden and matched them to 92 490 births to women without history of breast cancer during the same period. The mean time from breast cancer diagnosis to conception was 4.3 years, and from diagnosis to birth 5.1 years. The majority of breast cancer survivors (39.1%) were aged 35–39 years at delivery (Table 1). One-third of women (36.4%) were primiparous, a third (36.3%) had a second child, whereas the rest (27.3%) had three or more children (including the current birth). In addition, although the incidence of births in breast cancer survivors remained stable for most of the study period, an increasing trend was observed during the latest period (2008–2017). Most breast cancer survivors were born in the Nordic countries including Sweden (88.4%), and a large proportion had undergone postgraduate education (38.9%). Half of the breast cancer survivors who delivered in 1982 or later (53.4%) had a BMI in the normal range of 18.5–24.9 kg/m^2^, the majority did not smoke during pregnancy (82.9%) and experienced no infertility issues (89.2%). Healthy comparators predominantly showed similar distributions for the other characteristics to those of breast cancer survivors. Among women who gave birth 1995–2017, 5.4% of breast cancer survivors and 8.1% of comparators reported using fertility treatments to conceive.

**Table 1.** Characteristics of the study population of breast cancer survivors who had singleton births in 1973–2017.

Characteristic	Healthy comparators	All births to BC survivors	<1 year since BC diagnosis	1–<2 years since BC diagnosis	2–<3 years since BC diagnosis	3–<5 years since BC diagnosis	≥5 years since BC diagnosis
	N (%)	N (%)	N (%)	N (%)	N (%)	N (%)	N (%)
Total	92490 (100.0)	926 (100.0)	93 (100.0)	145 (100.0)	142 (100.0)	214 (100.0)	332 (100.0)
Age at diagnosis							
<30 years	NA	377 (40.7)	22 (23.7)	36 (24.8)	48 (33.8)	81 (37.9)	190 (57.2)
30–34 years	NA	343 (37.0)	32 (34.4)	50 (34.5)	52 (36.6)	96 (44.9)	113 (34.0)
35–39 years	NA	173 (18.7)	29 (31.2)	50 (34.5)	34 (23.9)	33 (15.4)	27 (8.1)
40–49 years	NA	33 (3.6)	10 (10.8)	9 (6.2)	8 (5.6)	4 (1.9)	2 (0.6)
Age at delivery							
<30 years	8214 (8.9)	80 (8.6)	14 (15.1)	17 (11.7)	17 (12.0)	19 (8.9)	13 (3.9)
30–34 years	26790 (29.0)	271 (29.3)	31 (33.3)	50 (34.5)	48 (33.8)	66 (30.8)	76 (22.9)
35–39 years	36309 (39.3)	362 (39.1)	33 (35.5)	47 (32.4)	50 (35.2)	96 (44.9)	136 (41.0)
≥40 years	21177 (22.9)	213 (23.0)	15 (16.1)	31 (21.4)	27 (19.0)	33 (15.4)	107 (32.2)
Birth order							
First child	33654 (36.4)	337 (36.4)	43 (46.2)	48 (33.1)	55 (38.7)	86 (40.2)	105 (31.6)
Second child	33536 (36.3)	336 (36.3)	23 (24.7)	55 (37.9)	53 (37.3)	73 (34.1)	132 (39.8)
Third or higher order	25300 (27.3)	253 (27.3)	27 (29.0)	42 (29.0)	34 (23.9)	55 (25.7)	95 (28.6)
Year of diagnosis							
1960–1977	NA	121 (13.1)	15 (16.1)	8 (5.5)	9 (6.3)	26 (12.1)	63 (19.0)
1978–1987	NA	177 (19.1)	24 (25.8)	29 (20.0)	28 (19.7)	39 (18.2)	57 (17.2)
1988–1997	NA	199 (21.5)	24 (25.8)	35 (24.1)	33 (23.2)	44 (20.6)	63 (19.0)
1998–2007	NA	273 (29.5)	15 (16.1)	35 (24.1)	34 (23.9)	66 (30.8)	123 (37.0)
2008–2017	NA	156 (16.8)	15 (16.1)	38 (26.2)	38 (26.8)	39 (18.2)	26 (7.8)
Year of delivery							
1973–1987	20873 (22.6)	209 (22.6)	37 (39.8)	30 (20.7)	28 (19.7)	48 (22.4)	66 (19.9)
1988–1997	18146 (19.6)	182 (19.7)	24 (25.8)	32 (22.1)	32 (22.5)	38 (17.8)	56 (16.9)
1998–2007	18981 (20.5)	190 (20.5)	15 (16.1)	35 (24.1)	23 (16.2)	47 (22.0)	70 (21.1)
2008–2017	34490 (37.3)	345 (37.3)	17 (18.3)	48 (33.1)	59 (41.5)	81 (37.9)	140 (42.2)
Maternal country of birth							
Nordics incl Sweden	77722 (84.0)	819 (88.4)	82 (88.2)	131 (90.3)	123 (86.6)	190 (88.8)	293 (84.0)
Other countries (excl the Nordics)	14768 (16.0)	107 (11.6)	11 (11.8)	14 (9.7)	19 (13.4)	24 (11.2)	39 (11.7)
Maternal education							
<10 years	9257 (10.0)	92 (9.9)	17 (18.3)	13 (9.0)	12 (8.5)	25 (11.7)	25 (7.5)
10–13 years	31578 (34.1)	305 (32.9)	32 (34.4)	51 (35.2)	53 (37.3)	72 (33.6)	97 (29.2)
<3 years of undergraduate	14643 (15.8)	163 (17.6)	17 (18.3)	36 (24.8)	27 (19.0)	36 (16.8)	47 (14.2)
3 years of undergraduate or higher	36003 (38.9)	360 (38.9)	24 (25.8)	43 (29.7)	49 (34.5)	81 (37.9)	163 (49.1)
Missing	1009 (1.1)	6 (0.6)	3 (3.2)	2 (1.4)	1 (0.7)	0 (0)	0 (0)
BMI (kg/m^2^)^a^							
<18.5	1641 (2.0)	19 (2.3)	4 (5.6)	1 (0.8)	4 (3.0)	4 (2.1)	6 (2.0)
18.5–24.9	42034 (50.8)	444 (53.4)	29 (40.3)	72 (54.1)	71 (53.8)	96 (50.3)	176 (57.9)
25–29.9	16386 (19.8)	173 (20.8)	13 (18.1)	29 (21.8)	31 (23.5)	37 (19.4)	63 (20.7)
>30	7145 (8.6)	55 (6.6)	5 (6.9)	8 (6.0)	10 (7.6)	17 (8.9)	15 (4.9)
Missing	15552 (18.8)	141 (16.9)	21 (29.2)	23 (17.3)	16 (12.1)	37 (19.4)	44 (14.5)
Smoking during pregnancy^a^							
No	68805 (83.1)	690 (82.9)	53 (73.6)	110 (82.7)	114 (86.4)	158 (82.7)	255 (83.9)
Yes	8969 (10.8)	81 (9.7)	11 (15.3)	14 (10.5)	14 (10.6)	17 (8.9)	25 (8.2)
Missing	4984 (6.0)	61 (7.3)	8 (11.1)	9 (6.8)	4 (3.0)	16 (8.4)	24 (7.9)
Infertility^a^							
No	70485 (85.2)	742 (89.2)	64 (88.9)	125 (94.0)	117 (88.6)	167 (87.4)	269 (88.5)
Yes	12 273 (14.8)	90 (10.8)	8 (11.1)	8 (6.0)	15 (11.4)	24 (12.6)	35 (11.5)
Fertility treatment^b^							
No	54 187 (91.9)	557 (94.6)	37 (97.4)	88 (95.7)	87 (92.6)	130 (94.9)	215 (94.3)
Yes	4806 (8.1)	32 (5.4)	1 (2.6)	4 (4.3)	7 (7.4)	7 (5.1)	13 (5.7)

BC, breast cancer; NA, not applicable.

Available for 1982–2017, missing for all births 1973–1981 (N = 9826, 10.5%).

Available for 1995–2017, missing for all births 1973–1994 (N = 33 834, 36.2%).

Information on tumor characteristics and treatment was available for years 1992–2017 (Supplementary Table S3). In total, 56.8% of women had a pT1 sized tumor and 37.1% had a pT2 sized tumor. There was no lymph node involvement in 66.7% of the women, whereas 26.8% had 1–3 metastatic axillary nodes (pN1). None of the women had distant metastasis at diagnosis. Information on ER and PR status was available for 71.9% of breast cancer survivors diagnosed 1992–2017, and 36.5% of those had HER2 status recorded. Of the women with known receptor status, 54.7% had ER-positive tumors, 48.4% PR-positive tumors, and 19.5% HER2-positive tumors. Among women with information on surgery, 57.6% of women had breast-conserving surgery and 42.2% mastectomy. Moreover, 62.8% of women received radiotherapy, 61.6% chemotherapy, and 23.8% endocrine therapy. Among the 212 women who had chemotherapy, 23 women (20.0%) conceived within the first 2 years after breast cancer diagnosis. Among the 123 women who did not receive chemotherapy, 44 (33.3%) conceived within 2 years. Among the 82 women who received endocrine therapy, 39 (47.5%) conceived within less than 5 years after diagnosis.

**Figure 1.**
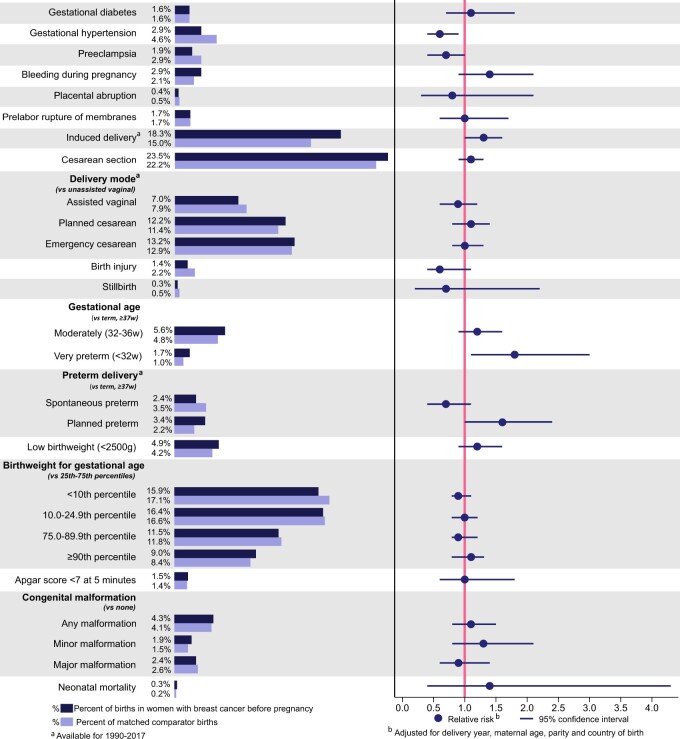
Obstetric and perinatal outcomes in breast cancer survivors 1973–2017 versus matched healthy comparators.

Compared to cancer-free comparators, women with a history of breast cancer before pregnancy had a higher risk of very preterm birth (RR; 1.85, 1.12–3.05), although the absolute risk was low in both groups (1.7% in women with a history of breast cancer and 1.0% in the matched comparators). Higher risks were also observed of induced delivery (RR; 1.27, 1.04–1.56), reflected by the sizeable difference in absolute risks between the two groups (18.3% and 15%, respectively). Moreover, women with a history of breast cancer had a higher risk of planned preterm birth (RR; 1.58, 1.04–2.40), and a lower risk of gestational hypertension (RR; 0.61, 0.42–0.90) (Fig. 1, exact RR estimates and crude absolute risk differences in Supplementary Table S4). There was also an indication of a higher risk of moderately preterm birth (RR, 1.19, 0.90–1.58), and bleeding during pregnancy (RR, 1.40, 0.95–2.06). Breast cancer before pregnancy was not associated with any other adverse obstetric or perinatal outcome. Further adjustment for maternal education, BMI and smoking during pregnancy, using multiple imputation to account for missing values of these covariates, had only minor effects on the associations presented in Fig. 1 (Supplementary Table S5).

There were only modest differences in associations with maternal cancer and birth outcomes between the two diagnosis periods 1960–2007 and 2008–2017 (Supplementary Table S6), except for induced delivery (*P*-value of interaction = 0.041) and congenital malformations (*P*-value of interaction = 0.043). The risk of induced delivery was higher among women with breast cancer diagnosed in 1960–1997 (RR; 1.72, 1.23–2.42) but not in those diagnosed in 1998–2016 (RR; 1.10, 0.86–1.41). The RR of congenital malformations was 1.35 (0.93–1.97) if the diagnosis was in the earlier period and 0.67 (0.37–1.22) in those diagnosed from 1998 to 2016. There was also an indication of a changing association for gestational hypertension (the effect estimate in the later period had half the magnitude of the one in the earlier period, from RR, 0.86, 0.53–1.40 to RR, 0.41, 0.22–0.77).

For women who conceived within 1 year after their breast cancer diagnosis, we observed a higher risk of cesarean section (RR; 1.69, 1.05–2.72), of very preterm birth (<32 w) (RR; 5.28, 1.88–14.85), and of low birthweight (RR; 2.68, 1.37–5.22) compared to healthy comparators (Table 2, crude absolute risk differences in Supplementary Table S7). For women who conceived during the second year after the breast cancer diagnosis, we found a higher risk of induced delivery (RR; 1.81, 1.13–2.90), cesarean section (RR; 1.49, 1.02–2.18), moderately preterm birth (32–36 weeks) (RR; 2.12, 1.21–3.71), and planned preterm birth (RR; 2.48, 1.07–5.72). No significantly higher risks of adverse obstetric or perinatal outcomes were observed for those who conceived more than 2 years after the breast cancer diagnosis.

**Table 2.** Select obstetric outcomes according to time between diagnosis of breast cancer and conception in breast cancer survivors, 1973–2017.

Outcome	Unexposed comparators, N (%)	0–1 years since BC diagnosis		1–2 years since BC diagnosis		2–3 years since BC diagnosis		3–5 years since BC diagnosis		5+ years since BC diagnosis		Lr test *P*-value^b^
		Exposed, N (%)	RR (95% CI)^a^	Exposed, N (%)	RR (95% CI)^a^	Exposed, N (%)	RR (95% CI)^a^	Exposed, N (%)	RR (95% CI)^a^	Exposed, N (%)	RR (95% CI)^a^	
Gestational hypertension												
No	88 270 (95.4)	90 (96.8)	1.00 (ref)	140 (96.6)	1.00 (ref)	139 (97.9)	1.00 (ref)	208 (97.2)	1.00 (ref)	322 (97.0)	1.00 (ref)	
Yes	4220 (4.6)	3 (3.2)	0.75 (0.23–2.40)	5 (3.4)	0.83 (0.34–2.05)	3 (2.1)	0.43 (0.14–1.37)	6 (2.8)	0.62 (0.27–1.41)	10 (3.0)	0.58 (0.31–1.09)	0.918
PROM												
No	90 891 (98.3)	91 (97.8)	1.00 (ref)	142 (97.9)	1.00 (ref)	139 (97.9)	1.00 (ref)	212 (99.1)	1.00 (ref)	326 (98.2)	1.00 (ref)	
Yes	1599 (1.7)	2 (2.2)	1.44 (0.35–5.92)	3 (2.1)	1.14 (0.36–3.60)	3 (2.1)	1.20 (0.38–3.80)	2 (0.9)	0.56 (0.14–2.27)	6 (1.8)	1.03 (0.46–2.32)	0.883
Induced delivery^c^												
No	56 332 (85.0)	38 (90.5)	1.00 (ref)	83 (78.3)	1.00 (ref)	88 (83.8)	1.00 (ref)	128 (2.1)	1.00 (ref)	200 (80.6)	1.00 (ref)	
Yes	9929 (15.0)	4 (9.5)	0.62 (0.22–1.75)	23 (21.7)	1.81 (1.13–2.90)	17 (16.2)	1.10 (0.65–1.87)	28 (17.9)	1.32 (0.87–2.01)	48 (19.4)	1.24 (0.90–1.71)	0.329
Cesarean section												
No	69 996 (77.8)	66 (71.7)	1.00 (ref)	104 (72.2)	1.00 (ref)	116 (83.5)	1.00 (ref)	161 (78.9)	1.00 (ref)	245 (75.4)	1.00 (ref)	
Yes	19 998 (22.2)	26 (28.3)	1.69 (1.05–2.72)	40 (27.8)	1.49 (1.02–2.18)	23 (16.5)	0.71 (0.45–1.13)	43 (21.1)	0.98 (0.69–1.39)	80 (24.6)	1.06 (0.82–1.38)	0.051
Delivery mode^c^												
Unassisted	44 552 (67.7)	32 (66.7)	1.00 (ref)	71 (65.7)	1.00 (ref)	77 (73.3)	1.00 (ref)	102 (68.5)	1.00 (ref)	163 (65.7)	1.00 (ref)	
Assisted vaginal	5208 (7.9)	4 (8.3)	0.97 (0.32–2.90)	6 (5.6)	0.83 (0.35–1.99)	9 (8.6)	0.97 (0.47–2.00)	12 (8.1)	0.95 (0.50–1.78)	15 (6.0)	0.80 (0.46–1.39)	0.992
Planned cesarean	7514 (11.4)	10 (20.8)	1.95 (0.93–4.09)	17 (15.7)	1.66 (0.95–2.88)	9 (8.6)	0.66 (0.32–1.34)	14 (9.4)	0.91 (0.51–1.60)	30 (12.1)	1.00 (0.67–1.49)	0.138
Emergency cesarean	8517 (12.9)	2 (4.2)	0.35 (0.08–1.52)	14 (13.0)	1.31 (0.72–2.39)	10 (9.5)	0.75 (0.38–1.47)	21 (14.1)	1.14 (0.70–1.88)	40 (16.1)	1.15 (0.80–1.65)	0.311
Gestational age												
≥37 weeks	87 021 (94.2)	83 (89.2)	1.00 (ref)	129 (89.0)	1.00 (ref)	131 (92.3)	1.00 (ref)	205 (95.8)	1.00 (ref)	310 (93.4)	1.00 (ref)	
Moderately preterm (32–36 weeks)	4437 (4.8)	6 (6.5)	1.36 (0.59–3.15)	14 (9.7)	2.12 (1.21–3.71)	8 (5.6)	1.18 (0.58–2.42)	8 (3.7)	0.77 (0.38–1.56)	16 (4.8)	1.04 (0.63–1.72)	0.231
Very preterm (<32 weeks)	887 (1.0)	4 (4.3)	5.28 (1.88–14.85)	2 (1.4)	1.39 (0.34–5.66)	3 (2.1)	2.71 (0.85–8.67)	1 (0.5)	0.45 (0.06–3.25)	6 (1.8)	1.91 (0.84–4.33)	0.146
Preterm delivery (planned versus spontaneous)^c^												
≥37 weeks	64 416 (94.3)	45 (91.8)	1.00 (ref)	100 (91.7)	1.00 (ref)	102 (94.4)	1.00 (ref)	154 (96.9)	1.00 (ref)	240 (94.1)	1.00 (ref)	
<37 weeks (spontaneous)	2392 (3.5)	2 (4.1)	1.20 (0.29–5.01)	3 (2.8)	0.84 (0.27–2.67)	1 (0.9)	0.26 (0.04–1.86)	4 (2.5)	0.70 (0.26–1.90)	6 (2.4)	0.68 (0.30–1.53)	0.746
<37 weeks (planned)	1475 (2.2)	2 (4.1)	1.88 (0.45–7.91)	6 (5.5)	2.48 (1.07–5.72)	5 (4.6)	2.32 (0.93–5.76)	1 (0.6)	0.28 (0.04–2.01)	9 (3.5)	1.68 (0.86–3.30)	0.121
Birthweight (crude)												
Normal (≥2500 g)	88 340 (95.8)	83 (89.2)	1.00 (ref)	138 (95.2)	1.00 (ref)	137 (96.5)	1.00 (ref)	206 (96.3)	1.00 (ref)	314 (95.4)	1.00 (ref)	
Low (<2500 g)	3877 (4.2)	10 (10.8)	2.68 (1.37–5.22)	7 (4.8)	1.11 (0.52–2.40)	5 (3.5)	0.87 (0.36–2.15)	8 (3.7)	0.92 (0.45–1.87)	15 (4.6)	1.11 (0.66–1.87)	0.213
Birthweight for gestational age (percentiles)												
<10.0	15 688 (17.1)	17 (18.3)	1.21 (0.67–2.19)	17 (11.8)	0.62 (0.36–1.05)	24 (16.9)	1.00 (0.62–1.60)	29 (13.6)	0.72 (0.48–1.10)	59 (18.1)	1.08 (0.80–1.48)	0.230
10.0–24.9	15 220 (16.6)	17 (18.3)	1.22 (0.68–2.21)	25 (17.4)	0.96 (0.61–1.51)	19 (13.4)	0.72 (0.43–1.21)	36 (16.8)	0.98 (0.67–1.44)	54 (16.6)	1.02 (0.74–1.40)	0.742
25.0–74.9	42 112 (46.0)	34 (36.6)	1.00 (ref)	77 (53.5)	1.00 (ref)	66 (46.5)	1.00 (ref)	104 (48.6)	1.00 (ref)	152 (46.6)	1.00 (ref)	
75.0–89.9	10 826 (11.8)	16 (17.2)	1.81 (0.99–3.31)	12 (8.3)	0.61 (0.33–1.13)	15 (10.6)	0.84 (0.48–1.48)	26 (12.1)	1.00 (0.65–1.55)	37 (11.3)	0.94 (0.65–1.35)	0.171
≥90.0	7652 (8.4)	9 (9.7)	1.55 (0.73–3.28)	13 (9.0)	0.96 (0.53–1.74)	18 (12.7)	1.57 (0.92–2.68)	19 (8.9)	1.02 (0.62–1.67)	24 (7.4)	0.83 (0.53–1.28)	0.361
Congenital malformations												
No	88 705 (95.9)	88 (94.5)	1.00 (ref)	137 (94.5)	1.00 (ref)	133 (93.7)	1.00 (ref)	207 (96.7)	1.00 (ref)	321 (96.7)	1.00 (ref)	
Yes	3785 (4.1)	5 (5.5)	1.24 (0.50–3.08)	8 (5.5)	1.39 (0.68–2.86)	9 (6.3)	1.60 (0.81–3.17)	7 (3.3)	0.81 (0.38–1.72)	11 (3.3)	0.80 (0.44–1.46)	0.497

BC, breast cancer; PROM, prelabour rupture of membranes; RR, relative risk of outcome reported as odds ratio (OR) for binary outcomes and relative risk ratio (RRR) for categorical outcomes.

Adjusted for calendar year, birth order, maternal age, and country of birth.

Likelihood-ratio test of interaction with time since breast cancer diagnosis.

Available for 1990–2017.

The effect of previous chemotherapy on adverse obstetric and perinatal outcomes was assessed in the subset of women diagnosed from 1992 and onwards (Table 3, crude absolute risk differences in Supplementary Table S8). Compared to cancer-free comparators, women who were treated with chemotherapy and who conceived <2 years after diagnosis had higher risks of planned cesarean section (RR 2.54, 1.13–5.72), moderately (RR; 3.59, 1.38–9.32) and very preterm birth (RR; 6.75, 1.51–30.09). No increased risks were observed for women treated with chemotherapy who conceived ≥2 years after diagnosis, and their risks of cesarean section (RR 0.55, 0.35–0.84) and low birthweight for gestational age (RR 0.56, 0.34–0.93) were lower than in comparator births. Among women who conceived ≥2 years after breast cancer diagnosis, a higher risk of induced delivery was observed only in women who had not received chemotherapy (RR 1.92, 1.18–3.15).

**Table 3.** Select obstetric and perinatal outcomes according to previous chemotherapy treatment in women who gave birth following breast cancer diagnosed in 1992–2017 compared to healthy comparators.

	BC without chemotherapy, conception <2 years after diagnosis			BC with chemotherapy, conception <2 years after diagnosis			Lr test
	Exposed, N (%)	Comparators, N (%)	RR (95% CI)^a^	Exposed, N (%)	Comparators, N (%)	RR (95% CI)^a^	*P*-value^b^
Induced delivery							
No	34 (81.0)	3686 (85.3)	1.00 (ref)	33 (80.5)	3670 (86.0)	1.00 (ref)	
Yes	8 (19.0)	635 (14.7)	1.36 (0.62–2.99)	8 (19.5)	599 (14.0)	1.52 (0.69–3.32)	0.847
Cesarean section							
No	29 (69.0)	3022 (71.8)	1.00 (ref)	30 (69.8)	3378 (80.6)	1.00 (ref)	
Yes	13 (31.0)	1186 (28.2)	1.18 (0.60–2.32)	13 (30.2)	811 (19.4)	1.85 (0.95–3.60)	0.353
Delivery mode							
Unassisted	27 (64.3)	2586 (61.5)	1.00 (ref)	28 (65.1)	3125 (74.7)	1.00 (ref)	
Assisted vaginal	2 (4.8)	431 (10.3)	0.50 (0.11–2.19)	2 (4.7)	248 (5.9)	0.76 (0.17–3.34)	0.689
Planned cesarean	7 (16.7)	591 (14.1)	1.15 (0.48–2.75)	8 (18.6)	366 (8.7)	2.54 (1.13–5.72)	0.192
Emergency cesarean	6 (14.3)	595 (14.2)	1.05 (0.41–2.68)	5 (11.6)	445 (10.6)	1.17 (0.44–3.11)	0.872
Gestational age							
Term (≥37 weeks)	43 (97.7)	4108 (93.7)	1.00 (ref)	36 (83.7)	4102 (95.4)	1.00 (ref)	
Moderately preterm (32–36 weeks)	1 (2.3)	224 (5.1)	0.43 (0.06–3.14)	5 (11.6)	161 (3.7)	3.59 (1.38–9.32)	0.027
Very preterm (<32 weeks)	0 (0.0)	53 (1.2)	NA	2 (4.7)	36 (0.8)	6.75 (1.51–30.09)	0.053
Birthweight for gestational age (percentiles)							
<10.0	8 (18.2)	742 (17.0)	1.23 (0.52–2.89)	3 (7.0)	675 (15.7)	0.35 (0.10–1.18)	0.084
10.0–24.9	5 (11.4)	718 (16.5)	0.79 (0.29–2.17)	6 (14.0)	730 (17.0)	0.69 (0.28–1.69)	0.843
25.0–74.9	17 (38.6)	2017 (46.3)	1.00 (ref)	25 (58.1)	2033 (47.4)	1.00 (ref)	
75.0–89.9	7 (15.9)	506 (11.6)	1.63 (0.67–3.98)	5 (11.6)	505 (11.8)	0.79 (0.30–2.10)	0.282
≥90.0	7 (15.9)	375 (8.6)	2.51 (1.01–6.24)	4 (9.3)	343 (8.0)	0.93 (0.32–2.73)	0.160
Congenital malformation							
No	42 (95.5)	4204 (95.8)	1.00 (ref)	41 (95.3)	4164 (96.8)	1.00 (ref)	
Yes	2 (4.5)	186 (4.2)	1.10 (0.26–4.62)	2 (4.7)	136 (3.2)	1.51 (0.36–6.31)	0.763

	BC without chemotherapy, conception ≥2 years after diagnosis			BC with chemotherapy, conception ≥2 years after diagnosis			Lr test
	Exposed, N (%)	Comparators, N (%)	**RR (95% CI)** ^a^	Exposed, N (%)	Comparators, N (%)	**RR (95% CI)** ^a^	*P*-value ^b^
Induced delivery							
No	62 (72.9)	7270 (83.6)	1.00 (ref)	140 (84.3)	13 921 (82.9)	1.00 (ref)	
Yes	23 (27.1)	1428 (16.4)	1.92 (1.18–3.15)	26 (15.7)	2868 (17.1)	0.90 (0.59–1.38)	0.023
Cesarean section							
No	60 (72.3)	6052 (71.6)	1.00 (ref)	138 (84.7)	12 300 (74.8)	1.00 (ref)	
Yes	23 (27.7)	2403 (28.4)	1.00 (0.61–1.65)	25 (15.3)	4136 (25.2)	0.55 (0.35–0.84)	0.071
Delivery mode							
Unassisted	54 (65.1)	5284 (62.6)	1.00 (ref)	128 (78.5)	10 965 (66.7)	1.00 (ref)	
Assisted vaginal	6 (7.2)	760 (9.0)	0.77 (0.32–1.86)	10 (6.1)	1331 (8.1)	0.63 (0.32–1.22)	0.722
Planned cesarean	9 (10.8)	1067 (12.6)	0.81 (0.39–1.67)	11 (6.7)	1923 (11.7)	0.49 (0.26–0.91)	0.314
Emergency cesarean	14 (16.9)	1336 (15.8)	1.00 (0.54–1.88)	14 (8.6)	2213 (13.5)	0.56 (0.32–0.99)	0.181
Gestational age							
Term (≥37 weeks)	86 (97.7)	8275 (94.1)	1.00 (ref)	164 (97.0)	15 965 (94.5)	1.00 (ref)	
Moderately preterm (32–36 weeks)	1 (1.1)	434 (4.9)	0.23 (0.03–1.64)	4 (2.4)	760 (4.5)	0.51 (0.19–1.39)	0.439
Very preterm (<32 weeks)	1 (1.1)	87 (1.0)	1.20 (0.17–8.70)	1 (0.6)	169 (1.0)	0.57 (0.08–4.10)	0.606
Birthweight for gestational age (percentiles)							
<10.0	19 (21.6)	1494 (17.1)	1.42 (0.81–2.47)	19 (11.2)	2885 (17.1)	0.56 (0.34–0.93)	0.016
10.0–24.9	16 (18.2)	1434 (16.4)	1.16 (0.64–2.08)	24 (14.2)	2884 (17.1)	0.76 (0.48–1.20)	0.276
25.0–74.9	41 (46.6)	4065 (46.6)	1.00 (ref)	86 (50.9)	7769 (46.2)	1.00 (ref)	
75.0–89.9	10 (11.4)	996 (11.4)	0.97 (0.48–1.96)	21 (12.4)	1944 (11.5)	0.99 (0.61–1.60)	0.969
≥90.0	2 (2.3)	735 (8.4)	0.27 (0.06–1.12)	19 (11.2)	1352 (8.0)	1.33 (0.80–2.20)	0.015
Congenital malformation							
No	87 (98.9)	8490 (96.5)	1.00 (ref)	164 (97.0)	16 281 (96.3)	1.00 (ref)	
Yes	1 (1.1)	310 (3.5)	0.31 (0.04–2.21)	5 (3.0)	619 (3.7)	0.80 (0.33–1.95)	0.344

BC, breast cancer; RR, relative risk of outcome reported as odds ratio (OR) for binary outcomes and relative risk ratio (RRR) for categorical outcomes.

Adjusted for calendar year, birth order, maternal age, and country of birth.

Likelihood-ratio test of interaction with treatment received.

## Discussion

In a population-based setting, we found that women who gave birth after breast cancer had significantly higher risks of having an induced delivery, very preterm birth, and planned preterm birth compared to women without previous cancer, but that was restricted to women who conceived within 2 years after diagnosis. Subgroup analysis further restricts the risk of preterm delivery to the women who received chemotherapy and conceived within 2 years after diagnosis. Women who conceived within 2 years of the breast cancer diagnosis also had higher risks of planned cesarean section and low birthweight. We did not observe any significantly higher risks among women who conceived more than 2 years after diagnosis.

A recent meta-analysis by Lambertini and colleagues also showed a higher risk of preterm birth among breast cancer survivors (Lambertini *et al.*, 2021). On the other hand, they found a higher risk of small for gestational age, which we did not find. One possible reason for this discrepancy is that the average time from diagnosis to delivery was shorter in the studies included in the meta-analysis.

Our findings that women who conceived less than 2 years after breast cancer diagnosis had higher risks of preterm birth corroborate the results by Hartnett *et al.* (2018). Further, we found that infants born to women who conceived within 1–2 years after diagnosis were more likely to be moderately preterm and either induced or delivered through planned cesarean section. In subset analysis, higher risks of preterm birth and planned cesarean section were observed in women who had received chemotherapy and conceived within 2 years of diagnosis.

In contrast to a previous Swedish study by Dalberg *et al.* (2006), we did not find a substantial elevation in the risk of congenital malformations among women conceiving after breast cancer in our more recent and larger study. The risk of malformations was slightly higher in births to women diagnosed with breast cancer in 1960–1997 compared to 1998–2016. These findings, alongside a decrease in the prevalence of major malformations over time, could indicate improved preventative measures, advances in breast cancer treatment, or changes in registration of malformations. We also found an inverse relation between conceiving after breast cancer and prevalence of gestational hypertension, which may reflect that women with previous breast cancer were, in some aspects, healthier than the randomly sampled comparators.

Previous research has shown that ovarian function is regained in 27–75% of reproductive-aged women who were administered chemotherapy agents (Yu *et al.*, 2010; Ruddy *et al.*, 2014; Abel *et al.*, 2021). However, immunosuppression persists months or even years after chemotherapy (Verma *et al.*, 2016), which is likely to explain the observed associations between chemotherapy received within 2 years of conception and adverse obstetric and perinatal outcomes, such as preterm birth and low birthweight (Black *et al.*, 2017; Hartnett *et al.*, 2018). Conception through fertility treatments is also a known risk factor for preterm birth (Qin *et al.*, 2017). As the use of fertility treatments was uncommon in our study, and lower among women with previous breast cancer than comparators, it is unlikely to explain the observed higher risk of preterm birth. In women with chemotherapy who conceived ≥2 years after diagnosis, we found a lower risk of cesarean section, both planned and emergency, and of low birthweight for gestational age. As chemotherapy affects fertility, this group of women has already overcome certain fertility challenges. It is plausible that they represent a subset of women with favorable reproductive health, potentially associated with a lower likelihood of needing a cesarean section.

The current Swedish treatment guidelines recommend 4–6 months of adjuvant chemotherapy for intermediate and high-risk tumors and 5–10 years of endocrine therapy for ER-positive tumors (Regionala cancercentrum i samverkan, 2023). Furthermore, patients are recommended a washout period of 6–12 months after chemotherapy and immunotherapy before attempting to conceive. This means that many young breast cancer patients are at risk of entering menopause before completing treatment. In the ongoing POSITIVE trial, 13% of young breast cancer patients opted to undergo shorter endocrine therapy than recommended because of fertility concerns and 2% changed or declined chemotherapy for the same reason (Ruggeri *et al.*, 2019). The first results of this clinical trial are promising, indicating no increased risk of recurrence at 3 years of follow-up in women interrupting endocrine treatment after 2 years to attempt pregnancy (Partridge *et al.*, 2023). However, several studies have shown that the risk of recurrence increases if endocrine therapy is discontinued early (Herk-Sukel *et al.*, 2010; Collin *et al.*, 2021). Women who wish to conceive after breast cancer need to weigh the risk of recurrence as well as the risk of adverse obstetrical outcomes when conceiving shortly after diagnosis or chemotherapy, against the risk of infertility. Among those prescribed endocrine therapy in our study, nearly half conceived within 5 years of diagnosis which indicates that they discontinued or deferred treatment. A separate analysis of outcomes in this group was not possible in our study owing to the small numbers (n = 39).

Our study has several strengths. First, we had access to 44 years of follow-up in a nationwide setting with over 900 births exposed to maternal breast cancer, as well as information on time of conception and data on treatment. The use of population-based registries with high coverage and quality ensured unbiased ascertainment of cancers, births, and the investigated outcomes (Barlow *et al.*, 2009, Cnattingius *et al.*, 2023). In addition, the matched cohort approach eliminated confounding from key factors such as age at delivery. Lastly, we included an exhaustive selection of obstetric and perinatal outcomes.

A number of limitations should be acknowledged. To begin with, we have assessed a broad range of obstetric and perinatal outcomes of varying prevalence. Hence, it is important to interpret the findings in relation to effect sizes and clinically meaningful effects. Further, we did not have information on the end date of treatment. We utilized time between the date of diagnosis and conception as a proxy, which does not fully capture the effect of time since the end of treatment. In addition, information on treatment mode was only available from 1992. Owing to the small number of women with complete breast cancer treatment information, it was not possible to assess the outcomes by multiple treatment modes or to disentangle the potential influences of each treatment. Since the women with previous breast cancer were diagnosed and treated over a long period, changes in treatments and recommendations are likely to have impacted on the childbearing patterns and adverse outcomes. The analysis stratified by calendar period of breast cancer diagnosis revealed no significant differences over time for most outcomes, with the exceptions of induced delivery and congenital malformations where a decreasing trend was observed. Finally, we did not have information on pregnancies that did not lead to birth. Therefore, it was not possible to assess the risk of miscarriage or abortions in this population.

In conclusion, although a recent history of breast cancer was associated with higher risks of preterm birth, no other increased risks were found. In addition, no increased risks were found in women who conceived later than 2 years after diagnosis. Since family building is important to many young breast cancer patients, these findings are particularly important to inform breast cancer survivors and clinicians about future reproductive outcomes.

## Supplementary Material

hoae027_Supplementary_Data

## Data Availability

The data underlying this article cannot be shared publicly due to ethical considerations relating to the privacy of individuals who participated in the study.

## References

[hoae027-B1] Abel MK , Wald K, Sinha N, Letourneau JM, Simbulan R, Mok-Lin E, Cedars MI, Rosen MP. Conception after chemotherapy: post-chemotherapy method of conception and pregnancy outcomes in breast cancer patients. J Assist Reprod Genet 2021;38:1755–1765. 33740176 10.1007/s10815-021-02133-0 PMC8324739

[hoae027-B2] Arnold M , Morgan E, Rumgay H, Mafra A, Singh D, Laversanne M, Vignat J, Gralow JR, Cardoso F, Siesling S et al Current and future burden of breast cancer: global statistics for 2020 and 2040. Breast 2022;66:15–23. 36084384 10.1016/j.breast.2022.08.010 PMC9465273

[hoae027-B3] Barlow L , Westergren K, Holmberg L, Talbäck M. The completeness of the Swedish Cancer Register—a sample survey for year 1998. Acta Oncol 2009;48:27–33. 18767000 10.1080/02841860802247664

[hoae027-B4] Black KZ , Nichols HB, Eng E, Rowley DL. Prevalence of preterm, low birthweight, and small for gestational age delivery after breast cancer diagnosis: a population-based study. Breast Cancer Res 2017;19:11. 28143580 10.1186/s13058-017-0803-z PMC5282806

[hoae027-B5] Cardoso F , Loibl S, Pagani O, Graziottin A, Panizza P, Martincich L, Gentilini O, Peccatori F, Fourquet A, Delaloge S et al; European Society of Breast Cancer Specialists. The European Society of Breast Cancer Specialists recommendations for the management of young women with breast cancer. Eur J Cancer Oxf Engl 1990 2012;48:3355–3377. 10.1016/j.ejca.2012.10.004 23116682

[hoae027-B6] Cnattingius S , Källén K, Sandström A, Rydberg H, Månsson H, Stephansson O, Frisell T, Ludvigsson JF. The Swedish Medical Birth Register during five decades: documentation of the content and quality of the register. Eur J Epidemiol 2023;38:109–120. 36595114 10.1007/s10654-022-00947-5 PMC9867659

[hoae027-B7] Collin LJ , Cronin-Fenton DP, Ahern TP, Goodman M, McCullough LE, Waller LA, Kjærsgaard A, Damkier P, Christiansen PM, Ejlertsen B et al Early discontinuation of endocrine therapy and recurrence of breast cancer among premenopausal women. Clin Cancer Res 2021;27:1421–1428. 33334905 10.1158/1078-0432.CCR-20-3974 PMC7925421

[hoae027-B8] Corsello G , Giuffrè M. Congenital malformations. J Matern Fetal Neonatal Med 2012;25:25–29. 22356564 10.3109/14767058.2012.664943

[hoae027-B9] Dalberg K , Eriksson J, Holmberg L. Birth outcome in women with previously treated breast cancer—a population-based cohort study from Sweden. PLoS Med 2006;3:e336. 16968117 10.1371/journal.pmed.0030336 PMC1564170

[hoae027-B10] Forouzanfar MH , Foreman KJ, Delossantos AM, Lozano R, Lopez AD, Murray CJL, Naghavi M. Breast and cervical cancer in 187 countries between 1980 and 2010: a systematic analysis. Lancet 2011;378:1461–1484. 21924486 10.1016/S0140-6736(11)61351-2

[hoae027-B11] Fredriksson I. *National Quality Register for Breast Cancer (NKBC)*, 2023. https://statistik.incanet.se/brostcancer/ (13 September 2023, date last accessed).

[hoae027-B12] Hartnett KP , Mertens AC, Kramer MR, Lash TL, Spencer JB, Ward KC, Howards PP. Pregnancy after cancer: does timing of conception affect infant health? Cancer 2018;124:4401–4407. 30403424 10.1002/cncr.31732 PMC7886368

[hoae027-B13] Herk-Sukel M V , Poll-Franse LVvd, Voogd AC, Nieuwenhuijzen GAP, Coebergh JWW, Herings RMC. Half of breast cancer patients discontinue tamoxifen and any endocrine treatment before the end of the recommended treatment period of 5 years: a population-based analysis. Breast Cancer Res Treat 2010;122:843–851. 20058066 10.1007/s10549-009-0724-3

[hoae027-B14] Hill DA , Friend S, Lomo L, Wiggins C, Barry M, Prossnitz E, Royce M. Breast cancer survival, survival disparities, and guideline-based treatment. Breast Cancer Res Treat 2018;170:405–414. 29569018 10.1007/s10549-018-4761-7 PMC6002943

[hoae027-B15] Jacob L , Kalder M, Arabin B, Kostev K. Impact of prior breast cancer on mode of delivery and pregnancy-associated disorders: a retrospective analysis of subsequent pregnancy outcomes. J Cancer Res Clin Oncol 2017;143:1069–1074. 28220257 10.1007/s00432-017-2352-3 PMC11818950

[hoae027-B16] Lambertini M , Blondeaux E, Bruzzone M, Perachino M, Anderson RA, Azambuja E D, Poorvu PD, Kim HJ, Villarreal-Garza C, Pistilli B et al Pregnancy after breast cancer: a systematic review and meta-analysis. J Clin Oncol 2021;39:3293–3305. 34197218 10.1200/JCO.21.00535

[hoae027-B17] Langagergaard V , Gislum M, Skriver MV, Nørgård B, Lash TL, Rothman KJ, Sørensen HT. Birth outcome in women with breast cancer. Br J Cancer 2006;94:142–146. 16306874 10.1038/sj.bjc.6602878 PMC2361078

[hoae027-B18] Lee HM , Kim BW, Park S, Park S, Lee JE, Choi YJ, Kim SY, Woo SU, Youn HJ, Lee I. Childbirth in young Korean women with previously treated breast cancer: The SMARTSHIP study. Breast Cancer Res Treat 2019;176:419–427. 31020470 10.1007/s10549-019-05244-6

[hoae027-B19] Lindström L , Ageheim M, Axelsson O, Hussain-Alkhateeb L, Skalkidou A, Wikström A-K, Bergman E. Swedish intrauterine growth reference ranges for estimated fetal weight. Sci Rep 2021;11:12464. 34127756 10.1038/s41598-021-92032-2 PMC8203766

[hoae027-B20] Löfgren L , Eloranta S, Krawiec K, Asterkvist A, Lönnqvist C, Sandelin K; Steering Group of the National Register for Breast Cancer. Validation of data quality in the Swedish National Register for Breast Cancer. BMC Public Health 2019;19:495. 31046737 10.1186/s12889-019-6846-6 PMC6498669

[hoae027-B21] Partridge AH , Niman SM, Ruggeri M, Peccatori FA, Azim HA, Colleoni M, Saura C, Shimizu C, Sætersdal AB, Kroep JR, et al; POSITIVE Trial Collaborators. Interrupting endocrine therapy to attempt pregnancy after breast cancer. N Engl J Med 2023;388:1645–1656. 37133584 10.1056/NEJMoa2212856 PMC10358451

[hoae027-B22] Qin J-B , Sheng X-Q, Wu D, Gao S-Y, You Y-P, Yang T-B, Wang H. Worldwide prevalence of adverse pregnancy outcomes among singleton pregnancies after in vitro fertilization/intracytoplasmic sperm injection: a systematic review and meta-analysis. Arch Gynecol Obstet 2017;295:285–301. 27896474 10.1007/s00404-016-4250-3

[hoae027-B23] Radecka B , Litwiniuk M. Breast cancer in young women. Ginekol Pol 2016;87:659–663. 27723074 10.5603/GP.2016.0062

[hoae027-B24] Regionala cancercentrum i samverkan. Nationellt vårdprogram bröstcancer. Version no. 4.3 [in Swedish]. Regionalt Cancercentrum Stockholm Gotland, 2023. https://kunskapsbanken.cancercentrum.se/diagnoser/brostcancer/vardprogram// [19 April 2023, date last accessed).

[hoae027-B25] Ruddy KJ , O’Neill A, Miller KD, Schneider BP, Baker E, Sparano JA, Dang C, Northfelt DW, Sledge GW, Partridge AH. Biomarker prediction of chemotherapy-related amenorrhea in premenopausal women with breast cancer participating in E5103. Breast Cancer Res Treat 2014;144:591–597. 24584876 10.1007/s10549-014-2891-0 PMC4334112

[hoae027-B26] Ruggeri M , Pagan E, Bagnardi V, Bianco N, Gallerani E, Buser K, Giordano M, Gianni L, Rabaglio M, Freschi A et al Fertility concerns, preservation strategies and quality of life in young women with breast cancer: baseline results from an ongoing prospective cohort study in selected European Centers. Breast 2019;47:85–92. 31362134 10.1016/j.breast.2019.07.001

[hoae027-B27] Santana DS , Surita FG, Cecatti JG. Multiple pregnancy: epidemiology and association with maternal and perinatal morbidity. Rev Bras Ginecol Obstet 2018;40:554–562. 30231294 10.1055/s-0038-1668117 PMC10316907

[hoae027-B28] Stan D , Loprinzi CL, Ruddy KJ. Breast cancer survivorship issues. Hematol Oncol Clin North Am 2013;27:805–827, ix. 23915746 10.1016/j.hoc.2013.05.005 PMC3903408

[hoae027-B29] Stensheim H , Klungsøyr K, Skjaerven R, Grotmol T, Fosså SD. Birth outcomes among offspring of adult cancer survivors: a population-based study. Int J Cancer 2013;133:2696–2705. 23729011 10.1002/ijc.28292

[hoae027-B30] Verma R , Foster RE, Horgan K, Mounsey K, Nixon H, Smalle N, Hughes TA, Carter CRD. Lymphocyte depletion and repopulation after chemotherapy for primary breast cancer. Breast Cancer Res 2016;18:10. 26810608 10.1186/s13058-015-0669-x PMC4727393

[hoae027-B31] White IR , Royston P, Wood AM. Multiple imputation using chained equations: issues and guidance for practice. Stat Med 2011;30:377–399. 21225900 10.1002/sim.4067

[hoae027-B32] Yu B , Douglas N, Ferin MJ, Nakhuda GS, Crew K, Lobo RA, Hershman DL. Changes in markers of ovarian reserve and endocrine function in young women with breast cancer undergoing adjuvant chemotherapy. Cancer 2010;116:2099–2105. 20187091 10.1002/cncr.25037 PMC3625425

