# Supplementary material for: Obstetric and perinatal outcomes in women with previous breast cancer: a nationwide study of singleton births 1973–2017
Source: Hum Reprod Open. 2024 May 4;2024(2):hoae027. doi: 10.1093/hropen/hoae027 (PMC11112047; doi:10.1093/hropen/hoae027)
Supplement: hoae027_Supplementary_Data [file hoae027_supplementary_data.docx]

Supplementary material


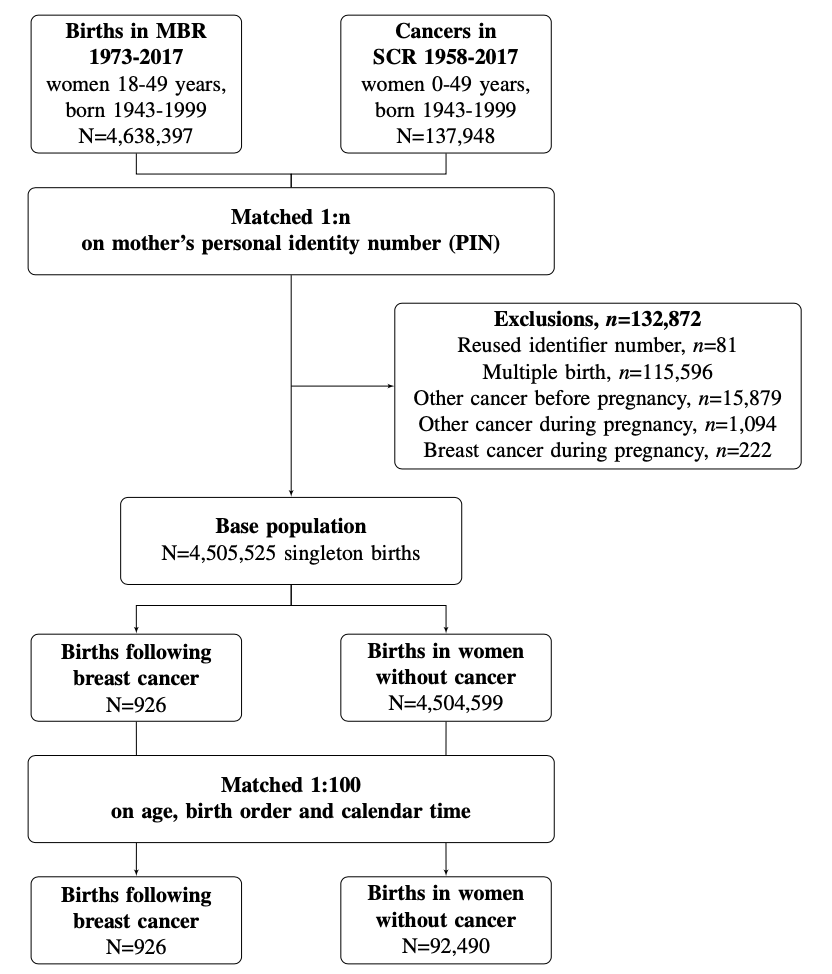


**Supplementary Figure S1.** Flowchart of study population. MBR Medical Birth Register, SCR Swedish Cancer Register.


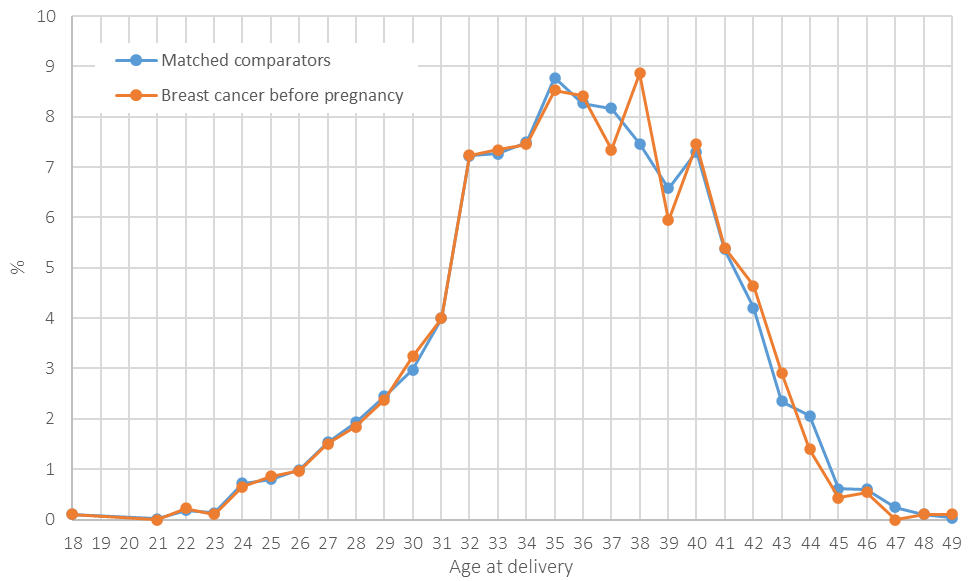


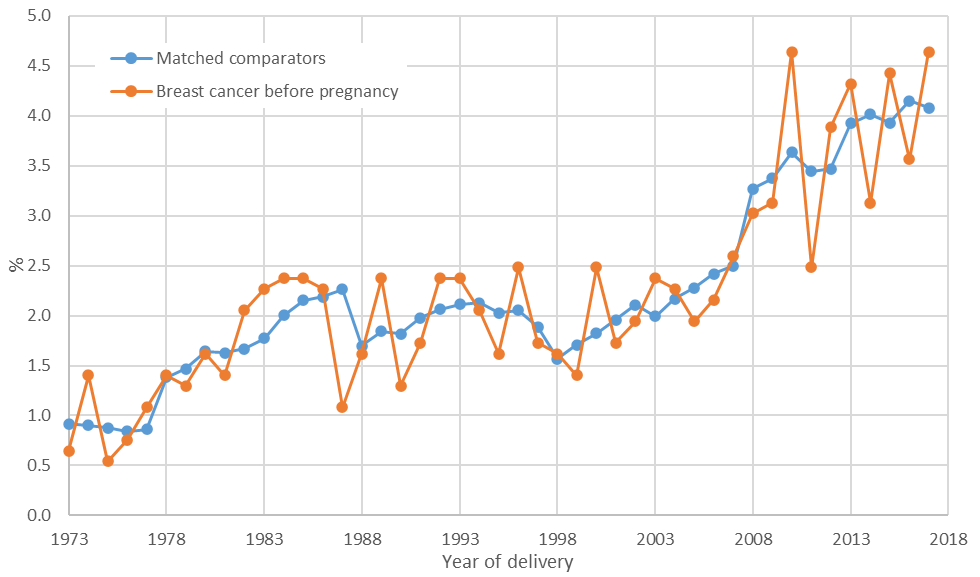


**Supplementary Figure S2.** Distribution of age at delivery (upper panel) and year of delivery (lower panel) among women with a diagnosis of breast cancer before pregnancy and matched comparators.

**Supplementary Table S1.** Diagnosis codes for identifying outcomes in the Medical Birth Register.

|  | **ICD8** | **ICD9** | **ICD10** |
| --- | --- | --- | --- |
| **Maternal diagnosis** |  |  |  |
| Gestational diabetes | 761,1 | 648A | O24 |
| Gestational hypertension | 637 | 642 | O13-O15 |
| Pre-eclampsia | 637,03–637,10 | 642E–642H | O14–O15 |
| Placental abruption | 632,10; 651,4 | 641C | O45 |
| Bleeding during pregnancy (and antepartum) | 632,2–632,4; 651,5 | 640; 641B; 641D; 641X | O20; O46; O67 |
| Prelabor rupture of membranes | 634,95; 661,0 | 658B | O42 |
| **Child diagnosis** |  |  |  |
| Birth injury | 772 | 767 | P10–P15 |
| Any congenital malformation | 740–759 | 740–759 | Q00–Q99 |
| Minor congenital malformation | 745,10; 745,39; 745,40; 745,83; 745,99; 747,09^a^; 747,70; 750,10; 752,10; 754,01; 754,02; 754,09; 755,61; 757,10; 757,19; 758,99 | 744B; 744E; 747A^a^; 747F; 750A; 752F; 754A; 754B; 754G; 757G; 758E | Q10.5; Q17.0–Q17.5; Q17.9; Q18.0; Q18.1; Q18.9; Q25.0^a^; Q27.0; Q32.0; Q38.1; Q38.2; Q52.3; Q52.5; Q53.0–Q53.2; Q53.9; Q62.7; Q65.3–Q65.6; Q66.1–Q66.9; Q67.0; Q67.4; Q67.8; Q68.0; Q75.2; Q79.1; Q79.9; Q82.5; Q89.9; Q95.0; Q95.9 |
| ^a^ Patent ductus arteriosus categorized as a minor malformation in children born preterm (gestational age <37 wks.).  Congenital malformations were categorized as major or minor using the European network of population-based registries for the epidemiological surveillance of congenital anomalies (EUROCAT) guide 1.3: <https://eu-rd-platform.jrc.ec.europa.eu/sites/default/files/EUROCAT-Guide-1.3.pdf>, accessed 16 November 2022 and the Swedish versions of the International Classification of Diseases [ICD] revisions 8, 9 and 10:  <https://www.socialstyrelsen.se/statistik-och-data/klassifikationer-och-koder/icd-10>, accessed 16 November 2022. | | | |

**Supplementary Table S2.** Missingness of outcomes in the study population.

|  | **Breast cancer before pregnancy N (%)** | **Healthy comparators**  **N (%)** |
| --- | --- | --- |
| **Gestational diabetes** | 0 (0.0) | 0 (0.0) |
| **Gestational hypertension** | 0 (0.0) | 0 (0.0) |
| **Preeclampsia** | 0 (0.0) | 0 (0.0) |
| **Bleeding during pregnancy** | 0 (0.0) | 0 (0.0) |
| **Placental abruption** | 0 (0.0) | 0 (0.0) |
| **Prelabor rupture of membrane** | 0 (0.0) | 0 (0.0) |
| **Induced delivery (1990**–**2017)** | 23 (3.4) | 2079 (3.0) |
| **Mode of delivery (1990**–**2017)** | 22 (3.2) | 2549 (3.7) |
| **Birth injury** | 0 (0.0) | 0 (0.0) |
| **Stillbirth** | 0 (0.0) | 0 (0.0) |
| **Caesarean section** | 22 (2.4) | 2496 (2.7) |
| **Preterm delivery** | 0 (0.0) | 145 (0.2) |
| **Preterm delivery (planned vs spontaneous, (1990**–**2017)** | 0 (0.0) | 57 (0.1) |
| **Birthweight (crude)** | 3 (0.3) | 273 (0.3) |
| **Birthweight (for gestational age)** | 7 (0.8) | 992 (1.1) |
| **Apgar score at 5 minutes** | 22 (2.4) | 3102 (3.4) |
| **Congenital malformation** | 0 (0.0) | 0 (0.0) |
| **Neonatal mortality** | 4 (0.4) | 472 (0.5) |

**Supplementary Table S3.** Tumour and treatment characteristics of women that gave birth after breast cancer diagnosed in 1992–2017.

|  | **N (%)** |
| --- | --- |
| **T pathological stage** |  |
| pT0 | 6 (1.2) |
| pT1 | 283 (56.8) |
| pT2 | 185 (37.1) |
| pT3 | 24 (4.8) |
| Missing | 64 |
| **N pathological stage** |  |
| pN0 | 336 (66.7) |
| pN1 | 135 (26.8) |
| pN2 | 27 (5.4) |
| pN3 | 6 (1.2) |
| Missing | 58 |
| **M clinical stage** |  |
| Negative | 466 (100.0) |
| Positive | 0 (0.0) |
| Missing | 96 |
| **Oestrogen receptor (ER) status** |  |
| Negative | 183 (45.3) |
| Positive | 221 (54.7) |
| Missing | 158 |
| **Progesterone receptor (PR) status** |  |
| Negative | 210 (51.6) |
| Positive | 197 (48.4) |
| Missing | 155 |
| **Human epidermal growth factor receptor 2 (HER2) status** |  |
| Negative | 165 (80.5) |
| Positive | 40 (19.5) |
| Missing | 357 |
| **Surgery** |  |
| Breast conserving surgery | 294 (57.6) |
| Mastectomy | 215 (42.2) |
| None | 1 (0.2) |
| Missing | 52 |
| **Radiotherapy** |  |
| No | 118 (37.2) |
| Yes | 199 (62.8) |
| Missing | 245 |
| **Chemotherapy** |  |
| No | 132 (38.4) |
| Yes | 212 (61.6) |
| Missing | 218 |
| **Endocrine therapy** |  |
| No | 262 (76.2) |
| Yes | 82 (23.8) |
| Missing | 218 |

**Supplementary Table S4.** Obstetric and perinatal outcomes in breast cancer survivors that gave birth 1973–2017 versus healthy comparators.

|  | **BC before pregnancy**  **N (%)** | **Healthy comparators**  **N (%)** | **Absolute risk difference**  **pp (95% CI)** | **Model 1  RR (95% CI) ^a^** | **Model 2  RR (95% CI) ^b^** |
| --- | --- | --- | --- | --- | --- |
| **Gestational diabetes** |  |  |  |  |  |
| No | 911 (98.4) | 91013 (98.4) | −0.0 (−0.8;0.8) | 1.00 (ref) | 1.00 (ref) |
| Yes | 15 (1.6) | 1477 (1.6) | +0.0 (−0.8;0.8) | 1.02 (0.61–1.70) | 1.10 (0.65–1.84) |
| **Gestational hypertension** |  |  |  |  |  |
| No | 899 (97.1) | 88270 (95.4) | +1.6 (0.6;2.7) | 1.00 (ref) | 1.00 (ref) |
| Yes | 27 (2.9) | 4220 (4.6) | −1.6 (−2.7; –0.6) | 0.62 (0.42–0.92) | 0.61 (0.42–0.90) |
| **Pre-eclampsia** |  |  |  |  |  |
| No | 908 (98.1) | 89804 (97.1) | 1.0 (0.1;1.9) | 1.00 (ref) | 1.00 (ref) |
| Yes | 18 (1.9) | 2686 (2.9) | −1.0 (−1.9;−0.1) | 0.66 (0.41–1.06) | 0.65 (0.41–1.05) |
| **Bleeding during pregnancy** |  |  |  |  |  |
| No | 899 (97.1) | 90508 (97.9) | −0.8 (−1.9;0.3) | 1.00 (ref) | 1.00 (ref) |
| Yes | 27 (2.9) | 1982 (2.1) | +0.8 (−0.3;1.9) | 1.38 (0.93–2.03) | 1.40 (0.95–2.06) |
| **Placental abruption** |  |  |  |  |  |
| No | 922 (99.6) | 91983 (99.5) | +0.1 (−0.3;0.5) | 1.00 (ref) | 1.00 (ref) |
| Yes | 4 (0.4) | 507 (0.5) | −0.1 (−0.5;0.3) | 0.79 (0.29–2.11) | 0.79 (0.29–2.11) |
| **Prelabor rupture of membranes** |  |  |  |  |  |
| No | 910 (98.3) | 90891 (98.3) | +0.0 (−0.8;0.8) | 1.00 (ref) | 1.00 (ref) |
| Yes | 16 (1.7) | 1599 (1.7) | −0.0 (−0.8;0.8) | 1.00 (0.61–1.64) | 1.01 (0.61–1.65) |
| **Induced delivery ^c^** |  |  |  |  |  |
| No | 537 (81.7) | 56332 (85.0) | −3.3 (−6.2;−0.3) | 1.00 (ref) | 1.00 (ref) |
| Yes | 120 (18.3) | 9929 (15.0) | +3.3 (0.3;6.2) | 1.27 (1.04–1.56) | 1.27 (1.04–1.56) |
| **Caesarean section** |  |  |  |  |  |
| No | 692 (76.5) | 69996 (77.8) | −1.2 (−4.0;1.5) | 1.00 (ref) | 1.00 (ref) |
| Yes | 212 (23.5) | 19998 (22.2) | +1.2 (−1.5;4.0) | 1.09 (0.93–1.28) | 1.10 (0.93–1.29) |
| **Delivery mode ^c^** |  |  |  |  |  |
| Unassisted vaginal | 445 (67.6) | 44552 (67.7) | −0.1 (−3.7;3.5) | 1.00 (ref) | 1.00 (ref) |
| Assisted vaginal | 46 (7.0) | 5208 (7.9) | −0.9 (−2.9;1.0) | 0.88 (0.64–1.21) | 0.88 (0.64–1.22) |
| Planned caesarean | 80 (12.2) | 7514 (11.4) | +0.7 (−1.8;3.2) | 1.07 (0.83–1.36) | 1.08 (0.84–1.37) |
| Emergency caesarean | 87 (13.2) | 8517 (12.9) | +0.3 (−2.3;2.9) | 1.03 (0.81–1.31) | 1.05 (0.82–1.33) |
| **Birth injury** |  |  |  |  |  |
| No | 913 (98.6) | 90468 (97.8) | +0.8 (0.0;1.5) | 1.00 (ref) | 1.00 (ref) |
| Yes | 13 (1.4) | 2022 (2.2) | −0.8 (−1.5;−0.0) | 0.64 (0.37–1.10) | 0.64 (0.37–1.11) |
| **Stillbirth** |  |  |  |  |  |
| No | 923 (99.7) | 92059 (99.5) | +0.1 (−0.2;0.5) | 1.00 (ref) | 1.00 (ref) |
| Yes | 3 (0.3) | 431 (0.5) | −0.1 (−0.5;0.2) | 0.69 (0.22–2.17) | 0.71 (0.23–2.21) |
| **Gestational age** |  |  |  |  |  |
| Term (≥37w) | 858 (92.7) | 87021 (94.2) | −1.6 (−3.3;0.1) | 1.00 (ref) | 1.00 (ref) |
| Moderately preterm (32–36w) | 52 (5.6) | 4437 (4.8) | +0.8 (−0.7;2.3) | 1.18 (0.89–1.57) | 1.19 (0.90–1.58) |
| Very preterm (<32w) | 16 (1.7) | 887 (1.0) | +0.8 (−0.1;1.6) | 1.83 (1.11–3.02) | 1.85 (1.12–3.05) |
| **Preterm delivery (planned or spontaneous)** **^c^** |  |  |  |  |  |
| Term (≥37w) | 641 (94.3) | 64416 (94.3) | −0.1 (−1.8;1.7) | 1.00 (ref) | 1.00 (ref) |
| <37w (Spontaneous) | 16 (2.4) | 2392 (3.5) | −1.2 (−2.3;−0.0) | 0.67 (0.41–1.10) | 0.68 (0.41–1.11) |
| <37w (Planned) | 23 (3.4) | 1475 (2.2) | +1.2 (−0.1;2.6) | 1.56 (1.02–2.37) | 1.58 (1.04–2.40) |
| **Birthweight (crude)** |  |  |  |  |  |
| Normal (≥2500g) | 878 (95.1) | 88340 (95.8) | −0.7 (−2.1;0.7) | 1.00 (ref) | 1.00 (ref) |
| Low (<2500g) | 45 (4.9) | 3877 (4.2) | +0.7 (−0.7;2.1) | 1.17 (0.86–1.58) | 1.18 (0.87–1.60) |
| **Birthweight for gestational age (percentiles)** |  |  |  |  |  |
| <10.0 | 146 (15.9) | 15688 (17.1) | −1.3 (−3.6;1.1) | 0.89 (0.74–1.08) | 0.91 (0.75–1.10) |
| 10.0–24.9 | 151 (16.4) | 15220 (16.6) | −0.2 (−2.6;2.2) | 0.96 (0.79–1.15) | 0.97 (0.80–1.17) |
| 25.0–74.9 | 433 (47.1) | 42112 (46.0) | +1.1 (−2.2;4.3) | 1.00 (ref) | 1.00 (ref) |
| 75.0–89.9 | 106 (11.5) | 10826 (11.8) | −0.3 (−2.4;1.8) | 0.95 (0.77–1.18) | 0.95 (0.77–1.18) |
| ≥90.0 | 83 (9.0) | 7652 (8.4) | +0.7 (−1.2;2.5) | 1.06 (0.84–1.35) | 1.06 (0.83–1.34) |
| **Apgar score at 5 minutes** |  |  |  |  |  |
| ≥7 | 890 (98.5) | 88047 (98.5) | −0.0 (−0.9;0.8) | 1.00 (ref) | 1.00 (ref) |
| <7 | 14 (1.5) | 1341 (1.5) | +0.0 (−0.8;0.9) | 1.03 (0.61–1.75) | 1.04 (0.61–1.77) |
| **Congenital malformations** |  |  |  |  |  |
| No | 886 (95.7) | 88712 (95.9) | −0.2 (−1.5;1.1) | 1.00 (ref) | 1.00 (ref) |
| Yes | 40 (4.3) | 3785 (4.1) | +0.2 (−1.1;1.5) | 1.06 (0.77–1.46) | 1.06 (0.77–1.45) |
| *Minor* | *18 (1.9)* | *1357 (1.5)* | +0.5 (−0.4;1.4) | 1.32 (0.82–2.11) | 1.32 (0.82–2.11) |
| *Major* | *22 (2.4)* | *2421 (2.6)* | −0.2 (−1.2;0.7) | 0.91 (0.60–1.39) | 0.91 (0.59–1.39) |
| **Neonatal mortality within 27 days** |  |  |  |  |  |
| No | 919 (99.7) | 91796 (99.8) | −0.1 (−0.5;0.3) | 1.00 (ref) | 1.00 (ref) |
| Yes | 3 (0.3) | 222 (0.2) | +0.1 −0.3;0.5) | 1.35 (0.43–4.22) | 1.36 (0.43–4.26) |
| BC; breast cancer. RR; Relative risk of outcome reported as odds ratio (OR) for binary outcomes and relative risk ratio (RRR) for categorical outcomes. **^a^** Conditioned for calendar year, birth order and maternal age.  **^b^** Conditioned for calendar year, birth order and maternal age, and adjusted for maternal country of birth.  **^c^** Available for 1990–2017.  Note: The percentages are directly interpretable as crude risks among the exposed women. Due to the matching, the percentages among comparators are the crude risk in a cancer-free population with the same age, calendar year and parity at delivery as the women with previous breast cancer. Absolute risk differences in percentage points (risk among exposed – unexposed) with 95% CI were calculated through unadjusted logistic regression and the postestimation command margins in Stata. | | | | | |

**Supplementary Table S5.** Obstetric and perinatal outcomes in breast cancer survivors that gave birth 1973–2017 versus healthy comparators, adjusted for maternal education, BMI and smoking during pregnancy using multiple imputation.

|  | **BC before pregnancy**  **N (%)** | **Healthy comparators N (%)** | **Model 2  RR (95% CI) ^a^** | **Model 3  RR (95% CI) ^b^** |
| --- | --- | --- | --- | --- |
| **Gestational diabetes** |  |  |  |  |
| No | 911 (98.4) | 90868 (98.4) | 1.00 (ref) | 1.00 (ref) |
| Yes | 15 (1.6) | 1477 (1.6) | 1.10 (0.65–1.84) | 1.12 (0.66–1.88) |
| **Gestational hypertension** |  |  |  |  |
| No | 899 (97.1) | 88136 (95.4) | 1.00 (ref) | 1.00 (ref) |
| Yes | 27 (2.9) | 4209 (4.6) | 0.62 (0.42–0.90) | 0.63 (0.43–0.93) |
| **Pre-eclampsia** |  |  |  |  |
| No | 908 (98.1) | 89665 (97.1) | 1.00 (ref) | 1.00 (ref) |
| Yes | 18 (1.9) | 2680 (2.9) | 0.65 (0.41–1.05) | 0.68 (0.42–1.09) |
| **Bleeding during pregnancy** |  |  |  |  |
| No | 899 (97.1) | 90368 (97.9) | 1.00 (ref) | 1.00 (ref) |
| Yes | 27 (2.9) | 1977 (2.1) | 1.40 (0.95–2.06) | 1.41 (0.95–2.07) |
| **Placental abruption** |  |  |  |  |
| No | 922 (99.6) | 91839 (99.5) | 1.00 (ref) | 1.00 (ref) |
| Yes | 4 (0.4) | 506 (0.5) | 0.79 (0.29–2.11) | 0.78 (0.29–2.10) |
| **Prelabor rupture of membranes** |  |  |  |  |
| No | 910 (98.3) | 90752 (98.3) | 1.00 (ref) | 1.00 (ref) |
| Yes | 16 (1.7) | 1593 (1.7) | 1.01 (0.61–1.66) | 1.02 (0.62–1.68) |
| **Induced delivery ^c^** |  |  |  |  |
| No | 537 (81.7) | 56289 (85.0) | 1.00 (ref) | 1.00 (ref) |
| Yes | 120 (18.3) | 9925 (15.0) | 1.27 (1.04–1.56) | 1.29 (1.05–1.58) |
| **Caesarean section** |  |  |  |  |
| No | 692 (76.5) | 69890 (77.8) | 1.00 (ref) | 1.00 (ref) |
| Yes | 212 (23.5) | 19964 (22.2) | 1.10 (0.94–1.29) | 1.11 (0.95–1.30) |
| **Delivery mode ^c^** |  |  |  |  |
| Unassisted vaginal | 445 (67.6) | 44518 (67.7) | 1.00 (ref) | 1.00 (ref) |
| Assisted vaginal | 46 (7.0) | 5206 (7.9) | 0.88 (0.64–1.21) | 0.88 (0.64–1.22) |
| Planned caesarean | 80 (12.2) | 7511 (11.4) | 1.08 (0.84–1.37) | 1.08 (0.85–1.38) |
| Emergency caesarean | 87 (13.2) | 8504 (12.9) | 1.05 (0.82–1.34) | 1.06 (0.84–1.36) |
| **Birth injury** |  |  |  |  |
| No | 913 (98.6) | 90327 (97.8) | 1.00 (ref) | 1.00 (ref) |
| Yes | 13 (1.4) | 2018 (2.2) | 0.64 (0.37–1.11) | 0.64 (0.37–1.11) |
| **Stillbirth** |  |  |  |  |
| No | 923 (99.7) | 91918 (99.5) | 1.00 (ref) | 1.00 (ref) |
| Yes | 3 (0.3) | 427 (0.5) | 0.71 (0.23–2.23) | 0.72 (0.23–2.25) |
| **Gestational age** |  |  |  |  |
| Term (≥37w) | 858 (92.7) | 87021 (94.2) | 1.00 (ref) | 1.00 (ref) |
| Moderately preterm (32–36w) | 52 (5.6) | 4437 (4.8) | 1.19 (0.90–1.58) | 1.21 (0.91–1.60) |
| Very preterm (<32w) | 16 (1.7) | 887 (1.0) | 1.85 (1.12–3.05) | 1.86 (1.12–3.07) |
| **Preterm delivery (planned or spontaneous)** **^c^** |  |  |  |  |
| Term (≥37w) | 641 (94.3) | 64416 (94.3) | 1.00 (ref) | 1.00 (ref) |
| <37w (Spontaneous) | 16 (2.4) | 2392 (3.5) | 0.68 (0.41–1.11) | 0.68 (0.41–1.12) |
| <37w (Planned) | 23 (3.4) | 1475 (2.2) | 1.58 (1.04–2.40) | 1.59 (1.04–2.42) |
| **Birthweight (crude)** |  |  |  |  |
| Normal (≥2500g) | 878 (95.1) | 88228 (95.8) | 1.00 (ref) | 1.00 (ref) |
| Low (<2500g) | 45 (4.9) | 3855 (4.2) | 1.19 (0.88–1.61) | 1.19 (0.88–1.62) |
| **Birthweight for gestational age (percentiles)** |  |  |  |  |
| <10.0 | 146 (15.9) | 15688 (17.1) | 0.91 (0.75–1.10) | 0.92 (0.76–1.11) |
| 10.0–24.9 | 151 (16.4) | 15220 (16.6) | 0.97 (0.80–1.17) | 0.96 (0.80–1.16) |
| 25.0–74.9 | 433 (47.1) | 42112 (46.0) | 1.00 (ref) | 1.00 (ref) |
| 75.0–89.9 | 106 (11.5) | 10826 (11.8) | 0.95 (0.77–1.18) | 0.96 (0.77–1.19) |
| ≥90.0 | 83 (9.0) | 7652 (8.4) | 1.06 (0.83–1.34) | 1.08 (0.85–1.38) |
| **Apgar score at 5 minutes** |  |  |  |  |
| ≥7 | 890 (98.5) | 87950 (98.5) | 1.00 (ref) | 1.00 (ref) |
| <7 | 14 (1.5) | 1334 (1.5) | 1.04 (0.61–1.78) | 1.06 (0.62–1.81) |
| **Congenital malformations** |  |  |  |  |
| No | 886 (95.7) | 88569 (95.9) | 1.00 (ref) | 1.00 (ref) |
| Yes | 40 (4.3) | 3776 (4.1) | 1.06 (0.77–1.45) | 1.06 (0.77–1.46) |
| *Minor* | 18 (1.9) | 1356 (1.5) | 1.32 (0.82–2.11) | 1.31 (0.82–2.10) |
| *Major* | 22 (2.4) | 2413 (2.6) | 0.91 (0.59–1.39) | 0.91 (0.60–1.40) |
| **Neonatal mortality within 27 days** |  |  |  |  |
| No | 919 (99.7) | 91662 (99.8) | 1.00 (ref) | 1.00 (ref) |
| Yes | 3 (0.3) | 215 (0.2) | 1.40 (0.45–4.39) | 1.41 (0.45–4.41) |
| BC; breast cancer. RR; Relative risk of outcome reported as odds ratio (OR) for binary outcomes and relative risk ratio (RRR) for categorical outcomes. **^a^** Conditioned for calendar year, birth order and maternal age, and adjusted for maternal country of birth. Corresponding to Model 2 in Figure 1 and Supplementary Table S4, after excluding observations missing gestational age (n=145, 0.2%).  **^b^** Conditioned for calendar year, birth order and maternal age, and adjusted for maternal country of birth, highest attained education, pre-pregnancy BMI and smoking during pregnancy. Observations missing gestational age (n=145, 0.2%) were excluded from all models. Multiple imputation by chained equations was used to account for missing values of education (1.1%), BMI (27.2%), smoking (15.8%). In total, 29.3% of births had incomplete information on at least one of these covariates. Education and BMI were imputed by multinomial logistic regression and smoking by logistic regression. The covariates were assumed to be missing at random given the other predictors in the imputation models: breast cancer before pregnancy, maternal country of birth, age at delivery, parity, calendar period, gestational age and infant congenital malformation, as well as education, BMI and smoking, respectively. Ten cycles of chained equations were used, and results from 50 imputation cycles were combined by Rubin’s rules.  **^c^** Available for 1990–2017. | | | | |

**Supplementary Table S6.** Obstetric and perinatal outcomes in breast cancer survivors according to year of breast cancer diagnosis.

|  | **BC diagnosis 1960**–**1997** | | | **BC diagnosis 1998**–**2016** | | |  |
| --- | --- | --- | --- | --- | --- | --- | --- |
|  | **Exposed**  **N (%)** | **Comparators**  **N (%)** | **RR**  **(95% CI) ^a^** | **Exposed**  **N (%)** | **Comparators**  **N (%)** | **RR**  **(95% CI) ^a^** | **p-value ^b^** |
| **Gestational diabetes** |  |  |  |  |  |  |  |
| No | 494 (99.4) | 49252 (99.3) | 1.00 (ref) | 417 (97.2) | 41761 (97.4) | 1.00 (ref) |  |
| Yes | 3 (0.6) | 348 (0.7) | 0.94 (0.30–2.98) | 12 (2.8) | 1129 (2.6) | 1.14 (0.64–2.04) | 0.764 |
| **Gestational hypertension** |  |  |  |  |  |  |  |
| No | 480 (96.6) | 47671 (96.1) | 1.00 (ref) | 419 (97.7) | 40599 (94.7) | 1.00 (ref) |  |
| Yes | 17 (3.4) | 1929 (3.9) | 0.86 (0.53–1.40) | 10 (2.3) | 2291 (5.3) | 0.41 (0.22–0.77) | 0.065 |
| **Pre-eclampsia** |  |  |  |  |  |  |  |
| No | 486 (97.8) | 48447 (97.7) | 1.00 (ref) | 422 (98.4) | 41357 (96.4) | 1.00 (ref) |  |
| Yes | 11 (2.2) | 1153 (2.3) | 0.94 (0.52–1.73) | 7 (1.6) | 1533 (3.6) | 0.44 (0.21–0.93) | 0.116 |
| **Bleeding during pregnancy** |  |  |  |  |  |  |  |
| No | 485 (97.6) | 48992 (98.8) | 1.00 (ref) | 414 (96.5) | 41516 (96.8) | 1.00 (ref) |  |
| Yes | 12 (2.4) | 608 (1.2) | 2.03 (1.13–3.64) | 15 (3.5) | 1374 (3.2) | 1.11 (0.66–1.87) | 0.136 |
| **Placental abruption** |  |  |  |  |  |  |  |
| No | 495 (99.6) | 49289 (99.4) | 1.00 (ref) | 427 (99.5) | 42694 (99.5) | 1.00 (ref) |  |
| Yes | 2 (0.4) | 311 (0.6) | 0.64 (0.16–2.58) | 2 (0.5) | 196 (0.5) | 1.02 (0.25–4.11) | 0.646 |
| **Prelabor rupture of membranes** |  |  |  |  |  |  |  |
| No | 489 (98.4) | 48810 (98.4) | 1.00 (ref) | 421 (98.1) | 42081 (98.1) | 1.00 (ref) |  |
| Yes | 8 (1.6) | 790 (1.6) | 1.02 (0.50–2.05) | 8 (1.9) | 809 (1.9) | 0.99 (0.49–2.01) | 0.966 |
| **Induced delivery ^c^** |  |  |  |  |  |  |  |
| No | 194 (82.2) | 20958 (88.9) | 1.00 (ref) | 343 (80.0) | 35374 (81.5) | 1.00 (ref) |  |
| Yes | 42 (17.8) | 2614 (11.1) | 1.72 (1.23–2.42) | 78 (18.2) | 7315 (18.1) | 1.10 (0.86–1.41) | 0.041 |
| **Caesarean section** |  |  |  |  |  |  |  |
| No | 393 (80.4) | 39126 (81.0) | 1.00 (ref) | 299 (72.0) | 30870 (74.0) | 1.00 (ref) |  |
| Yes | 96 (19.6) | 9151 (19.0) | 1.05 (0.83–1.32) | 116 (28.0) | 10847 (26.0) | 1.14 (0.92–1.43) | 0.592 |
| **Birth injury** |  |  |  |  |  |  |  |
| No | 486 (97.8) | 48196 (97.2) | 1.00 (ref) | 427 (99.5) | 42272 (98.6) | 1.00 (ref) |  |
| Yes | 11 (2.2) | 1404 (2.8) | 0.78 (0.43–1.42) | 2 (0.5) | 618 (1.4) | 0.32 (0.08–1.29) | 0.209 |
| **Stillbirth** |  |  |  |  |  |  |  |
| No | 494 (99.4) | 49361 (99.5) | 1.00 (ref) | 429 (100.0) | 42698 (99.6) | 1.00 (ref) |  |
| Yes | 3 (0.6) | 239 (0.5) | 1.27 (0.41–3.98) | 0 (0.0) | 192 (0.4) | NA | 0.062 |
| **Gestational age** |  |  |  |  |  |  |  |
| ≥37w | 454 (91.3) | 46503 (93.3) | 1.00 (ref) | 404 (94.2) | 40518 (94.5) | 1.00 (ref) |  |
| Moderately preterm (32–36w) | 32 (6.4) | 2514 (5.1) | 1.31 (0.91–1.87) | 20 (4.7) | 1923 (4.5) | 1.05 (0.67–1.64) | 0.450 |
| Very preterm (<32w) | 11 (2.2) | 453 (0.9) | 2.50 (1.36–4.58) | 5 (1.2) | 434 (1.0) | 1.18 (0.49–2.87) | 0.158 |
| **Birthweight (crude)** |  |  |  |  |  |  |  |
| Normal (≥2500g) | 466 (94.3) | 47208 (95.6) | 1.00 (ref) | 412 (96.0) | 41132 (95.9) | 1.00 (ref) |  |
| Low (<2500g) | 28 (5.7) | 2186 (4.4) | 1.31 (0.89–1.93) | 17 (4.0) | 1691 (3.9) | 1.02 (0.63–1.66) | 0.423 |
| **Birthweight for gestational age (percentiles)** |  |  |  |  |  |  |  |
| <10.0 | 85 (17.3) | 8463 (17.3) | 1.00 (0.77–1.29) | 61 (14.3) | 7225 (16.9) | 0.81 (0.61–1.09) | 0.299 |
| 10.0–24.9 | 82 (16.7) | 8071 (16.5) | 0.98 (0.76–1.27) | 69 (16.1) | 7149 (16.7) | 0.95 (0.72–1.25) | 0.852 |
| 25.0–74.9 | 228 (46.4) | 22194 (45.5) | 1.00 (ref) | 205 (47.9) | 19918 (46.6) | 1.00 (ref) |  |
| 75.0–89.9 | 53 (10.8) | 5866 (12.0) | 0.87 (0.64–1.18) | 53 (12.4) | 4960 (11.6) | 1.04 (0.77–1.41) | 0.423 |
| ≥90.0 | 43 (8.8) | 4193 (8.6) | 0.98 (0.70–1.37) | 40 (9.3) | 3459 (8.1) | 1.15 (0.82–1.63) | 0.509 |
| **Apgar score at 5 minutes** |  |  |  |  |  |  |  |
| ≥7 | 466 (98.1) | 46120 (98.6) | 1.00 (ref) | 424 (98.8) | 41927 (98.4) | 1.00 (ref) |  |
| <7 | 9 (1.9) | 658 (1.4) | 1.36 (0.70–2.65) | 5 (1.2) | 683 (1.6) | 0.73 (0.30–1.77) | 0.259 |
| **Congenital malformations** |  |  |  |  |  |  |  |
| No | 468 (94.2) | 47430 (95.6) | 1.00 (ref) | 418 (97.4) | 41275 (96.2) | 1.00 (ref) |  |
| Yes | 29 (5.8) | 2170 (4.4) | 1.35 (0.93–1.97) | 11 (2.6) | 1615 (3.8) | 0.67 (0.37–1.22) | 0.043 |
| BC; breast cancer. RR; Relative risk of outcome reported as odds ratio (OR) for binary outcomes and relative risk ratio (RRR) for categorical outcomes. **^a^** Adjusted for calendar year, birth order, maternal age and country of birth.  **^b^** Likelihood ratio test of interaction with calendar period of diagnosis.  **^c^** Available for births 1990–2017. | | | | | | | |

**Supplementary Table S7.** Absolute risk differences for select obstetric outcomes according to time between diagnosis of breast cancer and conception in breast cancer survivors 1973–2017.

|  | **0**–**1 years since  BC diagnosis** | | | **1**–**2 years since  BC diagnosis** | | | **2**–**3 years since  BC diagnosis** | | | **3**–**5 years since  BC diagnosis** | | | **≥5 years since  BC diagnosis** | | |
| --- | --- | --- | --- | --- | --- | --- | --- | --- | --- | --- | --- | --- | --- | --- | --- |
|  | **Exp %** | **Com**  **%** | **ARD**  **pp (95% CI)** ^a^ | **Exp**  **%** | **Com**  **%** | **ARD**  **pp (95% CI)** ^a^ | **Exp %** | **Com**  **%** | **ARD**  **pp (95% CI)** ^a^ | **Exp %** | **Com**  **%** | **ARD**  **pp (95% CI)** ^a^ | **Exp %** | **Com**  **%** | **ARD**  **pp (95% CI)** ^a^ |
| **Gestational hypertension** | 3.2 | 4.2 | −1.0 (−4.6;2.7) | 3.4 | 4.0 | −0.6 (−3.6;2.4) | 2.1 | 4.7 | −2.6 (−4.9;−0.2) | 2.8 | 4.4 | −1.6 (−3.8;0.7) | 3.0 | 5.0 | −2.0 (−3.8;−0.1) |
| **PROM** | 2.2 | 1.5 | +0.6 (−2.3;3.6) | 2.1 | 1.8 | +0.2 (−2.1;2.6) | 2.1 | 1.8 | +0.3 (−2.0;2.7) | 0.9 | 1.7 | −0.7 (−2.0;0.6) | 1.8 | 1.8 | +0.0 (−1.4;1.5) |
| **Induced delivery** ^b^ | 9.5 | 14.1 | −4.5 (−13.5;4.4) | 21.7 | 13.4 | +8.3 (0.4;16.2) | 16.2 | 14.8 | +1.4 (−5.7;8.4) | 17.9 | 14.3 | +3.6 (−2.4;9.7) | 19.4 | 16.3 | +3.0 (−1.9;8.0) |
| **Caesarean section** | 28.3 | 19.7 | +8.6 (−0.6;17.8) | 27.8 | 21.2 | +6.6 (−0.8;13.9) | 16.5 | 21.7 | −5.2 (−11.4;1.1) | 21.1 | 21.8 | −0.7 (−6.4;4.9) | 24.6 | 23.9 | +0.7 (−4.0;5.4) |
| **Delivery mode (vs unassisted vaginal)** ^b^ |  |  |  |  |  |  |  |  |  |  |  |  |  |  |  |
| Assisted vaginal | 8.3 | 9.1 | −0.8 (−8.6;7.1) | 5.6 | 7.2 | −1.7 (−6.0;2.7) | 8.6 | 8.2 | +0.4 (−5.0;5.8) | 8.1 | 8.3 | −0.2 (−4.6;4.2) | 6.0 | 7.6 | −1.6 (−4.6;1.4) |
| Planned caesarean | 20.8 | 10.6 | +10.2 (−1.3;21.8) | 15.7 | 10.5 | +5.2 (−1.7;12.1) | 8.6 | 11.4 | −2.8 (−8.2;2.6) | 9.4 | 10.7 | −1.3 (−6.0;3.4) | 12.1 | 12.4 | −0.3 (−4.4;3.8) |
| Emergency caesarean | 4.2 | 12.5 | −8.3 (−14.0;−2.6) | 13.0 | 11.5 | +1.4 (−4.9;7.8) | 9.5 | 12.6 | −3.1 (−8.7;2.6) | 14.1 | 12.6 | +1.5 (−4.1;7.1) | 16.1 | 14.0 | +2.1 (−2.5;6.7) |
| **Gestational age (vs term)** |  |  |  |  |  |  |  |  |  |  |  |  |  |  |  |
| Moderately preterm (32–36w) | 6.5 | 5.0 | +1.5 (−3.6;6.5) | 9.7 | 4.8 | +4.8 (0.0;9.7) | 5.6 | 4.9 | +0.7 (−3.1;4.5) | 3.7 | 4.8 | −1.1 (−3.6;1.5) | 4.8 | 4.7 | +0.1 (−2.2;2.4) |
| Very preterm (<32w) | 4.3 | 0.9 | +3.4 (−0.7;7.6) | 1.4 | 1.1 | +0.3 (−1.6;2.2) | 2.1 | 0.8 | +1.3 (−1.1;3.7) | 0.5 | 1.0 | −0.5 (−1.5;0.4) | 1.8 | 1.0 | +0.8 (−0.6;2.3) |
| **Preterm delivery (Planned vs spontaneous)** ^b^ |  |  |  |  |  |  |  |  |  |  |  |  |  |  |  |
| <37w (Spontaneous) | 4.1 | 3.6 | +0.5 (−5.0;6.1) | 2.8 | 3.4 | −0.6 (−3.7;2.5) | 0.9 | 3.7 | −2.7 (−4.6;−0.9) | 2.5 | 3.5 | −1.0 (−3.5;1.4) | 2.4 | 3.5 | −1.1 (−3.0;0.8) |
| <37w (Planned) | 4.1 | 2.2 | +1.9 (−3.7;7.4) | 5.5 | 2.3 | +3.2 (−1.0;7.5) | 4.6 | 2.0 | +2.6 (−1.3;6.6) | 0.6 | 2.2 | −1.5 (−2.8;−0.3) | 3.5 | 2.2 | +1.4 (−0.9;3.6) |
| **Low birthweight (<2500g)** | 10.8 | 4.4 | +6.4 (0.1;12.7) | 4.8 | 4.4 | +0.4 (−3.1;3.9) | 3.5 | 4.0 | −0.5 (−3.6;2.5) | 3.7 | 4.1 | −0.4 (−2.9;2.2) | 4.6 | 4.2 | +0.4 (−1.9;2.6) |
| **Birthweight for gestational age (percentiles)** |  |  |  |  |  |  |  |  |  |  |  |  |  |  |  |
| <10.0 | 18.3 | 18.7 | −0.4 (−8.3;7.5) | 11.8 | 16.6 | −4.8 (−10.1;0.5) | 16.9 | 16.9 | +0.0 (−6.2;6.2) | 13.6 | 17.3 | −3.7 (−8.3;0.9) | 18.1 | 17.0 | +1.1 (−3.1;5.3) |
| 10.0–24.9 | 18.3 | 17.5 | +0.8 (−7.1;8.6) | 17.4 | 16.0 | +1.4 (−4.9;7.6) | 13.4 | 17.0 | −3.6 (−9.3;2.0) | 16.8 | 16.4 | +0.4 (−4.6;5.4) | 16.6 | 16.6 | −0.1 (−4.1;4.0) |
| 75.0–89.9 | 17.2 | 11.5 | +5.7 (−2.0;13.4) | 8.3 | 12.2 | −3.9 (−8.4;0.7) | 10.6 | 12.2 | −1.6 (−6.7;3.5) | 12.1 | 11.5 | +0.7 (−3.7;5.1) | 11.3 | 11.9 | −0.5 (−4.0;2.9) |
| ≥90.0 | 9.7 | 7.7 | +2.0 (−4.0;8.0) | 9.0 | 8.4 | +0.6 (−4.1;5.3) | 12.7 | 8.2 | +4.4 (−1.1;9.9) | 8.9 | 8.4 | +0.5 (−3.3;4.3) | 7.4 | 8.6 | −1.2 (−4.1;1.6) |
| **Congenital malformation** | 5.4 | 4.4 | +1.0 (−3.6;5.6) | 5.5 | 4.0 | +1.5 (−2.2;5.2) | 6.3 | 4.0 | +2.3 (−1.7;6.3) | 3.3 | 4.0 | −0.7 (−3.1;1.7) | 3.3 | 4.1 | −0.8 (−2.7;1.1) |
| BC; breast cancer. Com; matched comparators. Exp; exposed. PROM; prelabor rupture of membranes. ARD; Absolute risk difference. ^a^ Adjusted for calendar year, birth order, maternal age, and country of birth.  ^b^ Available for 1990–2017.  Note: The percentages are directly interpretable as crude risks among the exposed women. Due to the matching, the percentages among comparators are the crude risk in a cancer-free population with the same age, calendar year and parity at delivery as the women with previous breast cancer. Absolute risk differences in percentage points (risk among exposed – unexposed) with 95% CI were calculated through unadjusted logistic regression and the postestimation command margins in Stata. | | | | | | | | | | | | | | | |

**Supplementary Table S8.** Absolute risk differences for select obstetric and perinatal outcomes according to previous chemotherapy treatment in women who gave birth following breast cancer diagnosed 1992–2017 compared to healthy comparators.

|  | **BC without chemotherapy,**  **conception <2 years after diagnosis** | | | **BC with chemotherapy,**  **conception <2 years after diagnosis** | | |
| --- | --- | --- | --- | --- | --- | --- |
|  | **Exposed %** | **Comparators %** | **ARD pp (95% CI)** | **Exposed %** | **Comparators %** | **ARD pp (95% CI)** |
| **Induced delivery** | 19.0 | 14.7 | +4.4 (−7.6;16.3) | 19.5 | 14.0 | +5.5 (−6.7;17.7) |
| **Caesarean section** | 31.0 | 28.2 | +2.8 (−11.3;16.8) | 30.2 | 19.4 | +10.9 (−2.9;24.7) |
| **Delivery mode (vs unassisted vaginal)** |  |  |  |  |  |  |
| Assisted vaginal | 4.8 | 10.3 | −5.5 (−12.0;1.0) | 4.7 | 5.9 | −1.3 (−7.6;5.1) |
| Planned caesarean | 16.7 | 14.1 | +2.6 (−8.7;13.9) | 18.6 | 8.7 | +9.9 (−1.8;21.5) |
| Emergency caesarean | 14.3 | 14.2 | +0.1 (−10.5;10.8) | 11.6 | 10.6 | +1.0 (−8.6;10.6) |
| **Gestational age (vs term)** |  |  |  |  |  |  |
| Moderately preterm (32–36w) | 2.3 | 5.1 | −2.8 (−7.3;1.6) | 11.6 | 3.7 | +7.9 (−1.7;17.5) |
| Very preterm (<32w) | 0.0 | 1.2 | −1.2 (NA) | 4.7 | 0.8 | +3.8 (−2.5;10.1) |
| **Birthweight for gestational age (percentiles)** |  |  |  |  |  |  |
| <10.0 | 18.2 | 17.0 | +1.2 (−10.3;12.6) | 7.0 | 15.7 | −8.8 (−16.5;−1.1) |
| 10.0–24.9 | 11.4 | 16.5 | −5.1 (−14.6;4.3) | 14.0 | 17.0 | −3.1 (−13.5;7.3) |
| 75.0–89.9 | 15.9 | 11.6 | +4.3 (−6.6;15.1) | 11.6 | 11.8 | −0.2 (−9.8;9.5) |
| ≥90.0 | 15.9 | 8.6 | +7.3 (−3.5;18.1) | 9.3 | 8.0 | +1.3 (−7.4;10.0) |
| **Congenital malformation** | 4.5 | 4.2 | +0.3 (−5.9;6.5) | 4.7 | 3.2 | +1.5 (−4.8;7.8) |
|  | **BC without chemotherapy,**  **conception ≥2 years after diagnosis** | | | **BC with chemotherapy,**  **conception ≥2 years after diagnosis** | | |
|  | **Exposed %** | **Comparators %** | **ARD pp (95% CI)** | **Exposed %** | **Comparators %** | **ARD pp (95% CI)** |
| **Induced delivery** | 27.1 | 16.4 | +10.6 (1.2;20.1) | 15.7 | 17.1 | −1.4 (−7.0;4.1) |
| **Caesarean section** | 27.7 | 28.4 | −0.7 (−10.4;9.0) | 15.3 | 25.2 | −9.8 (−15.4;−4.3) |
| **Delivery mode (vs unassisted vaginal)** |  |  |  |  |  |  |
| Assisted vaginal | 7.2 | 9.0 | −1.8 (−7.4;3.8) | 6.1 | 8.1 | −2.0 (−5.7;1.7) |
| Planned caesarean | 10.8 | 12.6 | −1.8 (−8.5;4.9) | 6.7 | 11.7 | −5.0 (−8.8;−1.1) |
| Emergency caesarean | 16.9 | 15.8 | +1.1 (−7.0;9.1) | 8.6 | 13.5 | −4.9 (−9.2;−0.5) |
| **Gestational age (vs term)** |  |  |  |  |  |  |
| Moderately preterm (32–36w) | 1.1 | 4.9 | −3.8 (−6.1;−1.5) | 2.4 | 4.5 | −2.1 (−4.4;0.2) |
| Very preterm (<32w) | 1.1 | 1.0 | +0.1 (−2.1;2.4) | 0.6 | 1.0 | −0.4 (−1.6;0.8) |
| **Birthweight for gestational age (percentiles)** |  |  |  |  |  |  |
| <10.0 | 21.6 | 17.1 | +4.5 (−4.2;13.1) | 11.2 | 17.1 | −5.9 (−10.7;−1.1) |
| 10.0–24.9 | 18.2 | 16.4 | +1.7 (−6.4;9.8) | 14.2 | 17.1 | −2.9 (−8.2;2.4) |
| 75.0–89.9 | 11.4 | 11.4 | −0.1 (−6.7;6.6) | 12.4 | 11.5 | +0.9 (−4.1;5.9) |
| ≥90.0 | 2.3 | 8.4 | −6.2 (−9.3;−3.0) | 11.2 | 8.0 | +3.2 (−1.6;8.0) |
| **Congenital malformation** | 1.1 | 3.5 | −2.4 (−4.6;−0.1) | 3.0 | 3.7 | −0.7 (−3.3;1.9) |
| ARD; Absolute risk difference.  Note: The percentages are directly interpretable as crude risks among the exposed women. Due to the matching, the percentages among comparators are the crude risk in a cancer-free population with the same age, calendar year and parity at delivery as the women with previous breast cancer. Absolute risk differences in percentage points (risk among exposed – unexposed) with 95% CI were calculated through unadjusted logistic regression and the postestimation command margins in Stata. | | | | | | |
